# *CHD-18 g*-modulated *Pseudomonas* taxa support poplar salt tolerance

**DOI:** 10.1093/ismejo/wrag138

**Published:** 2026-05-28

**Authors:** Yangwenke Liao, Qingyue Zhang, Jiafeng Zheng, Jinchi Zhang, Tingting Dai, Fuyuan Zhu, Víctor J Carrión, Manuel Delgado-Baquerizo, Christian Sonne, Fuliang Cao, Xiaogang Li

**Affiliations:** State Key Laboratory of Tree Genetics and Breeding, Nanjing Forestry University, Nanjing, 210037, Jiangsu, China; Co-Innovation Center for Sustainable Forestry in Southern China, Nanjing Forestry University, Nanjing, 210037, Jiangsu, China; State Key Laboratory of Tree Genetics and Breeding, Nanjing Forestry University, Nanjing, 210037, Jiangsu, China; Co-Innovation Center for Sustainable Forestry in Southern China, Nanjing Forestry University, Nanjing, 210037, Jiangsu, China; State Key Laboratory of Tree Genetics and Breeding, Nanjing Forestry University, Nanjing, 210037, Jiangsu, China; Co-Innovation Center for Sustainable Forestry in Southern China, Nanjing Forestry University, Nanjing, 210037, Jiangsu, China; Co-Innovation Center for Sustainable Forestry in Southern China, Nanjing Forestry University, Nanjing, 210037, Jiangsu, China; State Key Laboratory of Tree Genetics and Breeding, Nanjing Forestry University, Nanjing, 210037, Jiangsu, China; Co-Innovation Center for Sustainable Forestry in Southern China, Nanjing Forestry University, Nanjing, 210037, Jiangsu, China; State Key Laboratory of Tree Genetics and Breeding, Nanjing Forestry University, Nanjing, 210037, Jiangsu, China; Co-Innovation Center for Sustainable Forestry in Southern China, Nanjing Forestry University, Nanjing, 210037, Jiangsu, China; Departamento de Microbiología, Facultad de Ciencias, Campus Universitario de Teatinos s/n, Universidad de Málaga, Málaga, 29010, Málaga, Spain; Departamento de Protección de Cultivos, Instituto de Hortofruticultura Subtropical y Mediterránea La Mayora (IHSM-UMA-CSIC), Campus Universitario de Teatinos, Málaga, 29010, Málaga, Spain; Department of Microbial Ecology, Netherlands Institute of Ecology (NIOO-KNAW), Droevendaalsesteeg 10, Wageningen 6708 PB, The Netherlands; Institute of Biology, Leiden University, Sylviusweg 72, 2333 BE, Leiden, The Netherlands; Laboratorio de Biodiversidad y Funcionamiento Ecosistémico, Instituto de Recursos Naturales y Agrobiología de Sevilla (IRNAS), CSIC, Sevilla, 41012, Sevilla, Spain; Department of Ecoscience, Aarhus University; Frederiksborgvej 399, Roskilde DK-4000, Zealand, Denmark; Universidad UTE, Dirección General de Postgrados, SYNERA Hub, 170527, Quito, Ecuador; State Key Laboratory of Tree Genetics and Breeding, Nanjing Forestry University, Nanjing, 210037, Jiangsu, China; Co-Innovation Center for Sustainable Forestry in Southern China, Nanjing Forestry University, Nanjing, 210037, Jiangsu, China; State Key Laboratory of Tree Genetics and Breeding, Nanjing Forestry University, Nanjing, 210037, Jiangsu, China; Co-Innovation Center for Sustainable Forestry in Southern China, Nanjing Forestry University, Nanjing, 210037, Jiangsu, China

**Keywords:** root microbiota, salt stress, *Pseudomonas*, cinnamoyl-CoA hydratase/dehydrogenase, poplar

## Abstract

Against the background of global climate change, soil salinization has emerged as a major abiotic stressor constraining agroforestry productivity worldwide. Root-recruited microbes enhance plant stress resilience, and host–microbe interactions depend on plant root metabolism. Stress-tolerant plant genotypes exhibit a marked capacity to enrich beneficial root-associated microbes through specialized metabolic responses, thereby facilitating phenotypic plasticity. However, the molecular mechanisms underlying these plant-microbe interactions remain unclear. In this study, we compared salt tolerance among three poplar varieties under aseptic and non-aseptic conditions, and analyzed their rhizosphere bacterial community responses to salt stress. We found that microbial inoculation modulated poplar salt tolerance, and poplar shaped rhizosphere bacterial communities in a genotype-dependent manner. Transcriptome sequencing and targeted metabolomic analysis indicated that salt-tolerant poplar plants preferentially activate the phenylpropanoid biosynthesis pathway, accompanied by the enhanced root secretion of benzoic acid (BA) and salicylic acid (SA) and up-regulation of *CHD-18 g* encoding cinnamoyl-CoA hydratase/dehydrogenase. Overexpression of *CHD-18 g* increased rhizosphere *Pseudomonas* abundance by enhancing BA and SA biosynthesis. Binary interaction assays further showed that the BA-induced *Pseudomonas* taxa mitigated salt stress and promoted poplar growth under salt stress. Our findings propose a framework linking host gene expression, root metabolism, and key microbial taxa in conferring salt tolerance. This work uncovers a metabolic signaling mechanism by which trees shape their root microbiome to enhance stress adaptation, offering actionable genetic and ecological strategies for improving tree resilience in sustainable agroforestry systems.

## Introduction

Agroforestry maintains critical ecosystem services including food and timber production. Yet, plantations frequently experience abiotic stresses including soil salinization due to irrigation and fertilization [1]. Ionic toxicity from global salt-affected soils (>1 billion hectares) develops into osmotic stress, physiological dysfunction, and plant mortality, reducing productivity [2, 3]. Plant microbes are critical to enhance stress tolerance [4, 5], making it effective to take advantages of soil microbiomes to sustain agroforestry production under global change. Thereby, advancing our understanding on plant-microbe crosstalk under salinity is therefore paramount for supporting climate-resilient agroforestry.

Deciphering mechanisms behind host-microbe communication is essential to harness the potential of microbiomes to help hosts mitigate stress. However, our limited knowledge of how plant microbiomes are recruited hinders their practical application. Plants employ “cry for help” strategies to selectively recruit stress-alleviating microbiota supporting host stress resistance [6–8]. During this process, plants activate complex metabolic networks to communicate with soil microbes driving comprehensive changes in root microbiome [9–11]. Beneficial microbes, such as plant growth-promoting rhizobacteria and mycorrhizal fungi, are increasingly reported to be enriched in the root microbiome under stress conditions [12–15]. Until now, only a few salt-responsive taxa with their effects and functions have been particularly described, including bacteria belonging to *Bacillus* [16], *Pseudomonas* [7], *Streptomyces* [17], and *Rhizobiaceae* [18], as well as arbuscular mycorrhizal fungi [19]. However, we still know little about how plants recruit these beneficial microbes.

When characterizing the cry for help strategy in plant, the genetic and/or metabolic mechanisms that influence specific stress-induced microbial assembly to support host resistance remain unknown [20–22]. Emerging evidence supports the finding that root exudates (e.g. flavonoids, phytohormones, terpenes, and N-containing compounds) are important mediators facilitating beneficial plant-microbe interactions [23–25]. To date, previous studies have focused on identifying stress-induced metabolites in root exudates and exploring how these compounds recruit beneficial microbes [7, 26, 27]. Only a few works have reported the molecular regulation of the metabolic pathways involved in plant-microbe interaction, but most of them concentrated on plant defense [8, 28], or growth promotion under normal conditions [29, 30]. The detailed mechanisms by which plants reprogram root metabolism to recruit beneficial microbes to endure salt stress remain virtually unknown.

Poplar (*Populus* sp.) is globally planted for timber production and carbon sequestration, playing a critical role in economic and environmental sustainability [31]. Comprehensive genome databases and highly efficient systems for axenic culture and genetic transformation have been established for numerous *Populus* species, making poplar an ideal model for dissecting the molecular mechanisms underlying plant stress resistance [32]. Here we employed three poplar varieties, *Populus davidiana* × *P. bolleana* Loucne (SXY), *P. deltoides* × *Populus euramericana* “Nanlin 895” (NL895), and *P. alba* × *P. glandulosa* “84 K” (84 K), to explore the salt-tolerant mechanisms in plants. NL895 is from section (sect.) *Aigeiros* of *Populus*, and is widely cultivated in southern China [33]; SXY and 84 K belong to sect. *Leuce*, which are mainly distributed in northern China [34, 35]. *Leuce* species have been shown to exhibit higher salt tolerance than those from sect. *Aigeiros* [36].

We initially combined microbiota inoculation, high-throughput sequencing, targeted microbial isolation, and functional trait assays to identify root-recruited microbes and evaluate their contributions to host salt tolerance. Further, through integrated transcriptomic and metabolomic profiling coupled with genetic modification, we obtained molecular and metabolic evidence for plant-mediated regulation of the root microbiome under salt stress. These multi-dimensional experiments including microbe-metabolite pure-culture were combined to investigate the connection between plant gene, root metabolism, and the rhizosphere microbiomes under salt stress. We aimed to explore how plants -cry for help- to soil microbiota to fight environmental stress and its molecular regulation, providing tools to support agroforestry production by exploiting plant tolerant strategy in face of soil salinization.

## Methods and materials

### Preparation and transplanting of axenic poplar plantlets

Stem cuttings of SXY, NL895, and 84 K were surface-sterilized with 0.1% mercuric chloride for 8 min. The sterilized cuttings were subsequently cultured in vitro on Murashige and Skoog (MS) medium. Following sprouting, shoot tips (0.2 mm) were transferred to half-strength MS medium for subculturing to obtain endophyte-free seedlings. Detailed protocols for all culture and acclimation procedures are provided in the Supplementary Information (Materials and Methods).

### Mixed substrate culture experiments

**Figure 1:**
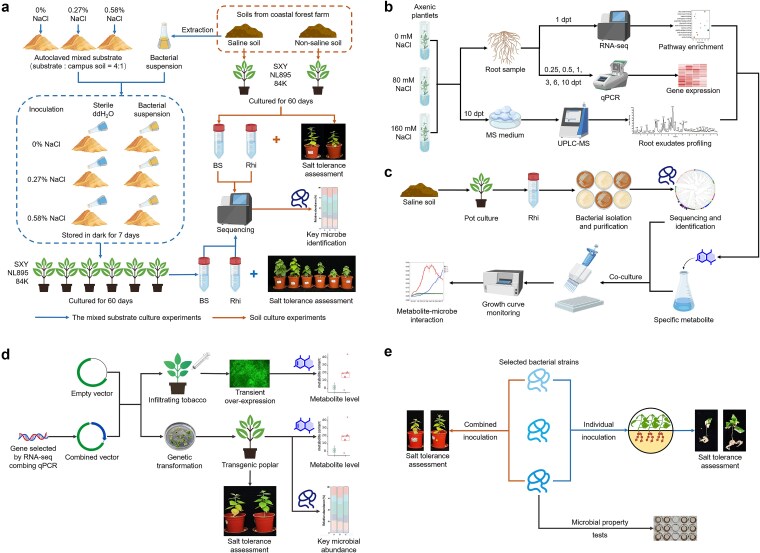
An overview of the experimental workflow for the present study. (**a**) identification of key microbial taxa via mixed substrate culture and soil culture experiments. BS, bulk soil sample; Rhi, rhizosphere soil sample. (**b**) Investigation of key metabolites and the critical regulatory genes. MS medium, Murashige and Skoog medium; RNA-seq, RNA sequencing; UPLC-MS, ultra-performance liquid chromatography-orbitrap-mass spectrum. (**c**) Microbial isolation and purification, and their interaction with the key metabolite. (**d**) Functional validation of gene that dominates biosynthesis of the key metabolite through transient over-expression and genetic transformation. (**e**) Investigation of effect from the metabolite-induced microbial taxa on host salt tolerance and microbial beneficial property tests.

The pot experiments were conducted following the workflow (Fig. 1a). Saline soils used for bacterial suspension extraction and soil culture experiment were obtained from the saline plot in Dongtai forest farm (32°51′8”N, 120°19′9″E) close to the coast of Jiangsu Province. The saline soil contained 0.33% (w/w) NaCl, a concentration non-lethal for poplar (Supplementary Table 1). The bacterial suspension used to inoculate the sterilized mixed substrate was then prepared as described in SI Materials and Methods.

For the mixed substrate culture, nutrient substrate (Xiangzheng, Hunan, China) was mixed thoroughly with soil from Nanjing Forestry University campus (v/v = 4:1; clay loam with salinity shown in Supplementary Table 1). For salt treatment, 50 ml of 9% and 14% NaCl solutions was separately added to each liter of the mixed substrate, resulting in final concentrations of 4.5 g/kg and 7 g/kg, respectively. The control group was established by supplementing 50 ml of sterile ddH₂O as replacement. Three groups of mixed substrate were then sealed in sterilization bags (BKMAN LAB, Changde, China) and autoclaved at 120°C for 40 min. After 30 days, due to the loss of salt adhering to the sterilization bag, the final salt content was determined as 0% (w/w; control), 0.27% (low salinity), and 0.58% (high salinity). The prepared bacterial suspension (sterile ddH_2_O as control) was then carefully inoculated to the sterilized mixed substrate of each salinity level as described in SI Materials and Methods, and stored in dark for 7 days for acclimation.

For each treatment, six plantlets of each variety were individually transplanted into sterilized plastic pots (sprayed with 75% alcohol) that contained 1 kg of the prepared mixed substrate. Plants were carefully watered once with sterile ddH_2_O every 3 days, ensuring no potential water leakage from the bottom of the pots. Photography, biomass measurement, and response value calculation were conducted as described in SI Materials and Methods.

### Soil culture experiments

For soil culture, six plantlets of each variety were transplanted into sterilized plastic pots filled with a 1 kg saline soil-perlite (4:1) mix (each pot containing only one plantlet). Control was established using non-saline soil (Supplementary Table 1) collected from a normal plot at the same site where the saline soil was obtained for replacement. Each pot was placed in an individual plastic tray for irrigation. Photographs were taken and biomasses of shoot and root were measured both at the 30th and 60th day post treatment (dpt).

### Sampling of soil and mixed substrate and amplicon sequencing

Rhizosphere soil was collected as previously described [37]. The plantlet in each pot was uprooted along with the surrounding soil. Excess soil was removed from roots by manually shaking, leaving an ~2-mm thick layer of soil still attached to roots. To collect root suspension, plant roots were placed in a 250 ml conical flask containing 100 ml of sterile PBS (autoclaved at 120°C for 20 min) and shaken at 220 rpm for 30 min at 4°C. Following removal of roots from the conical flasks, the suspension was centrifuged at 6000 × *g* for 10 min at 4°C. After discarding the supernatant, the rhizosphere soil was stored at −80°C until subsequent DNA extraction. Soil from pots without plantlets was sampled as bulk soil. The mixed substrate sampling was conducted using the same method.

Total DNA was extracted, and then the 16S rRNA gene samples were sequenced using the Illumina Novaseq 6000-PE250 System (Illumina Inc., San Diego, CA, USA) at Shanghai Personal Biotechnology Co., Ltd. (Shanghai, China). The protocols are as described in SI Materials and Methods.

### Sterile experimental design, root sampling, and exudates collection

To avoid effect of soil microbial activities on plant endogenous response to salt stress, we performed quantitative PCR (qPCR) and phenolic acid profiling using axenic plantlets (Fig. 1b). Axenic poplar plantlets of 4 cm height were directly transferred to semi-solid 1/2 MS medium with 2.5% sucrose, 0.19% agar (low level to avoid mechanical damages to roots during sampling), and 80 mM NaCl, with ddH_2_O as control. Plantlets were cultured under controlled condition as followed: a 16/8 h light/dark period, illumination intensity of 360 μmol m^−2^ s^−1^ and day/night temperatures of 23/23°C, with only one plantlet in each culture bottle.

Root tissues were collected at 1/4, 1/2, 1, 3, 6, and 10 dpt, respectively. For each treatment, three plantlets were randomly selected from independent culture bottles and carefully uprooted from the medium. Equal amounts of root tissues from each plantlet were pooled, thoroughly washed, cut into 1 cm segments, and homogenized to constitute one biological replicate. Samples were frozen in liquid nitrogen and stored in the refrigerator at −80°C for subsequent tests. MS medium within 1 cm from roots was collected at 10 dpt. Medium pooled from six plantlets in each treatment was fully mixed. Subsequently, 6 g of the mixed medium was dispensed in self-sealing bags as one biological replicate, and stored at −80°C for phenolic acid profiling. Three biological replicates were used for this assay.

### Root RNA extraction and real-time qPCR

Root tissues of axenic plantlets collected at 1 dpt were used for transcriptome analysis. Total mRNA extraction, cDNA library construction and RNA sequencing were performed. Transcriptomic analysis was completed by Personal Biotechnology Co., Ltd. (Shanghai, China). Detailed procedures are presented in the supplementary Materials and Methods.

qPCR was performed using roots of axenic plantlets collected at 0.25, 0.5, 1, 3, 6, and 10 dpt for verification of mRNA sequencing and investigation of benzoic acid (BA) and salicylic acid (SA) biosynthesis as previously described [38]. Primers were designed according to the reference genome (Supplementary Table 8), and qPCR was performed using the SYBR Green PCR MasterMix (Vazyme, Nanjing, China). The cycling conditions and expression level calculation were as described in SI Materials and Methods.

### Phenolic acids profiling

Phenolic acids profiling was performed through high-performance liquid chromatographic (HPLC)-mass spectrum (MS)-based targeted metabolome analysis as previously described [39], with minor modifications. Samples of MS medium for phenolic acid profiling were collected at 10 dpt and pretreated according to the protocol detailed in the SI Materials and Methods. The extracts were then analyzed using an ultra-performance liquid chromatography (UPLC)-Orbitrap-Mass Spectrum (MS) system (UPLC, Vanquish; MS, QE). The analytical conditions and the ESI source parameters were provided in SI Materials and Methods.

### Rhizosphere bacterial isolation, purification, and sequencing

Microbial isolation and purification, along with subsequent interaction assays involving the key metabolite, were performed following the workflow (Fig. 1c). The procedures of rhizosphere bacterial isolation, purification, and sequencing are detailed in SI Materials and Methods.

### Microbe-metabolite interaction and carbon source assay

To select BA-enriched strains, the isolated bacterial strains were first cultivated in TSB (soya peptone 0.5%, NaCl 0.5%, casein peptone 1.5%, pH 7.3) at 26°C, 160 rpm for 24 h, and then centrifuged at 6000 rpm, 4°C for 10 min. The supernatant was discarded, and the resulting cell pellets were resuspended and diluted with sterile ddH₂O to OD_600_ = 0.1. Aliquots (20 μl) of the adjusted bacterial suspension were then combined with 180 μl of 1/10 TSB supplemented with sodium benzoate at final concentrations of 0, 0.001, 0.05, 0.1, 1, 10, 25, and 50 mM. The mixtures were transferred to 96-well ELISA plates and incubated at 26°C, 160 rpm. The OD_600_ were measured at 48 and 72 h post inoculation, and the strains most induced by sodium benzoate as well as the most effective dose were both selected.

To evaluate the role of BA as a potential nutrient source supporting bacterial proliferation, resuspended bacterial cultures (20 μl, OD_600_ = 0.1) were co-cultured in carbon-free 1/10 TSB medium (0.15% tryptone, 0.05% NaCl) containing sodium benzoate at concentrations of 0, 1, 10, and 25 mM. Cultures were incubated at 26°C, 160 rpm [40]. The initial OD_600_ was recorded, after which absorbance was measured every 12 h until Day 7. All co-culture treatments were performed with four biological replicates.

### Vector construction, transient expression in tobacco, and transgenic poplar plants

Functional validation of the key phenolic acid biosynthetic gene – *cinnamoyl-CoA hydratase/dehydrogenase-18 g* (*CHD-18 g*; Podel.18G080700) – from SXY (termed *PdaCHD-18 g*) was conducted (Fig. 1d). To transiently express *PdaCHD-18 g* in tobacco leaves, coding sequence (CDS) was cloned into pCambia-1300 (laboratory stock) recombing *GFP* (primers shown in Supplementary Table 8), using the restriction enzymes SalI and BamHI from New England Biolabs, Inc. (NEB, Ipswich, MA, USA). The recombinant pCambia-1300 plasmid were transformed into *Escherichia coli* DH5α (Weidi Biotechnology Co., Ltd., Shanghai, China) by heat shock, and positive colonies were sequenced. The recombinant pCambia-1300 plasmid carrying *GFP*-tagged *PdaCHD-18 g* was introduced into *Agrobacterium tumefaciens* GV3101 (Weidi) by electroporation. Infiltration of tobacco leaves was performed as described in SI Materials and Methods.

To over-express *PdaCHD-18 g* in SXY, CDS was amplified and PCR products were ligated to pMDC32 plasmid (primers shown in Supplementary Table 8), with the restriction enzyme sites for AscI and SpeI (NEB). The recombinant pMDC32 plasmid (laboratory stock) were then transformed into DH5α, sequenced, and introduced into GV3101 as described before. Transformation of poplar was performed as described in SI Materials and Methods.

### Quantification of BA in plant tissues

To quantify BA content in plant tissues, HPLC was conducted as follow. BA in plant tissues was extracted as described in SI Materials and Methods. Chromatographic analysis of the prepared extracts was performed using an Agilent 1200 LC system (Agilent, Palo Alto, CA, USA), equipped with an HC-C18 reversed-phase column (250 × 4.6 mm, 5 μm; Agilent) and a variable wavelength detector (VWD; Agilent). The mobile phase was composed of 0.02 M ammonium acetate (solvent A) and methanol (solvent B) (v/v = 80:20). The analytical conditions were described in SI Materials and Methods.

### Phenotypic validation

Axenic poplar plantlets (WT and transgenic plants) were adapted as described before. Afterwards, 12 plantlets of each variety were transplanted into sterilized plastic pots filled with a 0.5 kg non-saline soil-perlite (4:1) mix (one plantlet for each pot) under the same culture conditions, and watered every 3 days ensuring no potential water leakage from the bottom of the pots.

After 20 days culture, salt treatment was conducted by spraying 25 ml of 1% NaCl solution around the surface of culture soil. Next, 25 ml of 2%, and 25 ml of 3% NaCl solution was added with a 10-day interval. The total content of NaCl reached 3.0 g kg^−1^. Plants were watered with sterile H_2_O after salt treatment immediately, and then with a 3-day interval, ensuring no potential water leakage from the bottom of the pots. At the 55th day post transplanting (the 35th day post salt treatment), leaf tissues and rhizosphere soil samples were collected as mentioned above, with plant biomass measured. Total chlorophyll of the poplar leaves was detected as described in SI Materials and Methods.

### *Pseudomonas* strains colonization

Effect of phenolic acid-induced *Pseudomonas* isolates and their combined inoculum on poplar salt tolerance was investigated (Fig. 1e). Three *Pseudomonas* strains, S51, S58, and K8, were selected because they were strongly induced by BA in pure-culture experiments. To test their effect on plant under salinity, S51, S58, and K8 were shaken in TSB at 160 rpm, 26°C for 48 h, and then centrifuged at 6000 rpm, 4°C for 10 min. The cell pellet was resuspended with sterile ddH_2_O, and OD_600_ was adjusted to 1.0 to obtain the bacterial suspension for inoculation. Axenic poplar plantlets of 3 cm height were transferred to solid 1/2 MS medium containing 0.6% agar and 80 mM NaCl, with three plantlets per petri dish. Root systems were affixed to medium. The culture was exposed to controlled condition as follow: a 16/8 h light/dark period, illumination intensity of 360 μmol m^−2^ s^−1^, and day/night temperatures of 26/26°C, and continued for 2 days (for phenolic acids exudation) [41]. Bacterial suspension (500 μl, OD_600_ = 1.0) was added dropwise to the roots of each plantlet, with sterile ddH_2_O inoculation as control (mock; Supplementary Fig. 18).

### Combined inoculation of BA-induced *Pseudomonas* isolates

To prepare the combined inoculum, the BA-induced *Pseudomonas* isolates, S51, S58, and K8, were activated at 30°C and 160 rpm, and the concentration of bacteria solution was adjusted to OD_600_ = 0.1 with sterile ddH_2_O. The individually prepared bacterial suspensions of S51, S58, and K8 were then mixed according to 1:1:1 (v:v:v) to obtain the combined inoculum. On the day axenic SXY plantlets were transplanted into sterilized non-saline soil and again at the 10th day post transplanting, 30 ml of combined inoculum was added to six plantlets of each variety at the joint of shoot and root. At the 20th day post transplanting, the inoculum volume was increased to 45 ml to achieve a comparable effect on the larger root system. An equal amount of sterile ddH_2_O was added to the six parallel pots as mock at each time point. Salt treatment was conducted as the same protocol as phenotypic validation for transgenic poplar plants. After 60 days culture, phenotypes were captured, and biomass as well as chlorophyll content were determined as above mentioned.

### Bacterial IAA production determination

IAA produced by bacterial strains was quantified following a previous study [42], with moderate modifications. The protocol is provided in SI Materials and Methods.

### Statistical analyses

For plant physiological assays, analysis of phenolic acid level and chlorophyll content was performed in three independent replicates, whereas the microbial properties were analyzed based on six replicates. The significance of differences among samples was determined using SPSS V17.0. The 16S rRNA gene sequencing data were analyzed using *R* v.4.4.1, and figures were generated using OriginPro (10.1.0.178), MEGA 11, and web-based tools. Detailed analyses were conducted as described in SI Materials and Methods.

## Results

### Rhizosphere microbiome mediates poplar salt tolerance

**Figure 2:**
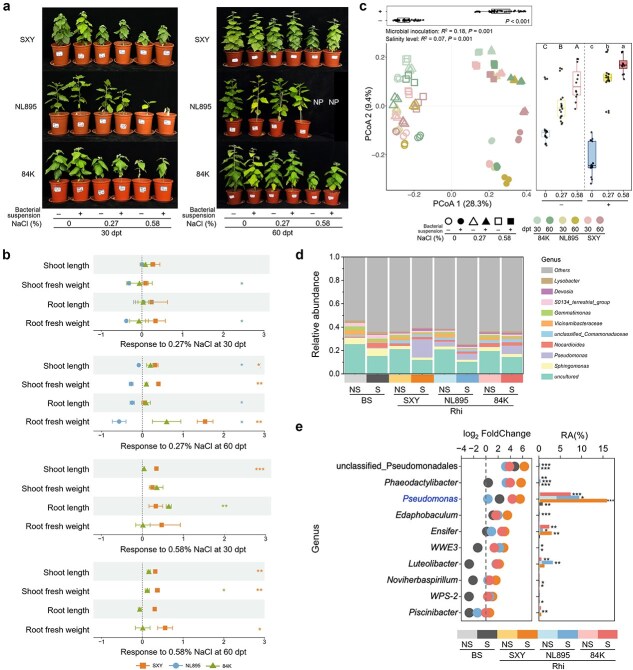
The salt-tolerant poplar enriches rhizosphere *Pseudomonas* under salt stress. (**a**) Effect of soil microbiota on poplar salt tolerance. NP, no photograph. “+”, the sterilized mixed substrate with bacterial suspension, and “-”, without bacterial suspension (mock); dpt, days post treatment. (**b**) Response of plant growth to bacterial suspension inoculation under salt stress. Mean ± SD; n = 3 plantlets; and significance was determined based on the differences in each trait between the bacterial suspension-treated and non-treated groups within each poplar variety under salt stress (^*^P < .05, ^**^P < .01, and ^***^  *P* < .001; ANOVA, Student’s t test). The left asterisks at the end of each line stand for significance of differences in 84K, the middle ones for NL895, and the right ones for SXY. (**c**) PCoA of rhizobacterial communities based on the Bray–Curtis dissimilarity metric (PERMANOVA). The box plots depict the distribution of data-point projections on the PCoA axes, where the horizontal line within each box represents the median, and the lower and upper bounds of the box indicate the 25th and 75th percentiles, respectively. The *P* value shown in the upper box plots is derived from Student’s t test comparing microbial community composition between bacterial inoculated and non-inoculated soil samples. The box plots on the right show on PCoA 2; uppercase and lowercase letters indicate significant differences across salinity levels within non-inoculated and inoculated soil samples, respectively (*P* < .05; ANOVA, Duncan’s test). (**d**) Relative abundance (RA) of the top 10 most abundant genera of rhizobacterial community from poplar cultured in saline soil at 60 dpt. NS, non-saline soil (control); S, saline soil (salt stress); BS, bulk soil; Rhi, rhizosphere. (**e**) The top 10 most enriched genera that SXY recruited more than the other varieties at 60 dpt. Enrichment of these genera in poplar rhizosphere by salt stress are visualized with dot plot (left). Genera whose RA was 0 in the control samples from poplar rhizosphere were discarded. The mean RA of each genus in different treatments is shown with bar plot (right). n = 3 biologically independent samples, and significances were calculated between NS and S group within each variety (^*^P < .05, ^**^P < .01, and ^***^P < .001; ANOVA, Student’s t test).

In a set of axenic culture experiments using three poplar varieties (SXY, NL895, and 84 K), we observed no discernible differences in responses of growth parameters upon salt stress (Supplementary Fig. 1). To determine whether soil microbiota can confer salt tolerance to poplar, we grew poplar in NaCl-treated sterilized mixed substrate, and inoculated plantlets with bacterial suspensions extracted from natural saline soil (Supplementary Table 1). We discovered that microbial colonization caused contrasting effects on poplar varieties (Fig. 2a), resulting in the highest salt tolerance in SXY but the lowest in NL895. In detail, SXY exhibited the strongest positive responses of growth parameters to microbial inoculation, especially in root biomasses, with the highest increases in survival rate (Supplementary Fig. 2), whereas NL895 showed negative responses (Fig. 2b). This shows the important role of soil microbiota for the establishment of poplar salt tolerance.

To clarify whether distinct responses to salt stress across poplar genotypes are associated to microbial community composition, we further investigated the overall divergences in their rhizosphere bacterial communities following microbial inoculation (Supplementary Table 2). Principal co-ordinates analysis (PCoA) revealed that rhizobacterial communities were separated along PCoA 1 by microbial inoculation and along PCoA 2 by salinity (Fig. 2c). Permutational multivariate analysis of variance (PERMANOVA) verified that both microbial inoculation (*R*^2^ = 0.18, *P* = .001) and salinity (*R*^2^ = 0.07, *P* = .001) significantly impacted community structure, with microbial inoculation exhibiting a stronger effect. Moreover, salt treatments resulted in more pronounced community shifts in inoculated soils than in non-inoculated soils (ANOVA; Duncan’s test, *P* < .05). Thus, PCoA was further performed on rhizosphere bacterial communities of inoculated soils under each salinity level (Supplementary Fig. 3). We observed that rhizosphere bacterial community assembly was primarily driven by sampling time (stress duration) and host genotype (PERMANOVA; control, *R*^2^ = 0.69, *P* = .001; low salinity, *R*^2^ = 0.73, *P* = .001; high salinity, *R*^2^ = 0.70, *P* = .001). Considering that both salinity and stress duration reflect salt stress degree, our results indicate that the tripartite interplay between microbial inoculation, salt stress degree, and plant genetic background integrates into a deterministic factor in microbial assemblies, resulting in correspondence of distinct microbial profiles to different salt-tolerant phenotypes.

To clarify whether these genotype-dependent microbial effects on host salt tolerance persist in soil, we conducted parallel culture experiments using non-saline and saline soils. The results mirrored the inoculation experiments: compared with plantlets grown in non-saline soil, NL895 exhibited the greatest decrease in shoot biomasses and root fresh weight in saline soil across all tested varieties. Furthermore, SXY showed less biomass reduction than 84 K at 60 dpt, especially in shoot and root fresh weight (Supplementary Fig. 4A and B). Collectively, SXY exhibited better performance than 84 K under salt stress, whereas NL895 showed the strongest growth-inhibition.

We performed an in-depth analysis of microbial variation among poplar genotypes grown in non-saline and saline soils (Supplementary Table 3). Salinity and host genotypes governed the assembly of rhizosphere bacterial community, and the influence of plant genotype became more pronounced as salt stress persisted (Supplementary Fig. 5A). Moreover, SXY displayed the most reduction in Shannon and Simpson index, indicating decreased diversity and evenness of rhizosphere bacterial community (Supplementary Fig. 5B). The relative abundance of *Pseudomonas* was elevated across all varieties under salt stress (Fig. 2d), with 48-fold, 19-fold, and 26% increases observed in the rhizosphere of SXY, 84 K, and NL895, respectively (Supplementary Table 4). These results imply selective bacterial recruitment by poplar roots under salt stress. Collectively, each poplar variety assembles a distinct rhizosphere bacterial community from the other two under salt stress, potentially through the enrichment of specific taxa.

To further identify the SXY-recruited bacterial taxa that support the best performance under salt stress, we examined the top 10 most enriched genera that SXY recruited more than other varieties in saline soils. *Pseudomonas* achieved the highest abundance (16%) among the top 10 most enriched genera (Fig. 2e and Supplementary Table 5), indicating substantial recruitment of *Pseudomonas* by SXY roots under salt stress. Further, a total of 14 biomarkers in the SXY-enriched amplicon sequence variants (ASVs) were identified as *Pseudomonas*, therein ASV 165091, ASV 285334, and ASV 249432 were the most abundant taxa (Supplementary Fig. 5C and Supplementary Table 6). Taken together, the most salt-tolerant variety SXY preferentially enriches specific *Pseudomonas* taxa in the rhizosphere under salt stress.

### Salt stress induces phenolic acid secretion in poplar roots

But how does poplar manipulate rhizosphere microbiome under salt stress? To address this question, we next examined the early transcriptional responses underlying metabolic changes. To avoid interference from root-associated microbiota, we exposed axenic plantlets to salt stress and analyzed their root transcriptome profiles (Supplementary Table 7). Principal component analysis showed that root gene expression profiles were separated mainly by genotype along axis 1 and by salinity along axis 2 (Supplementary Fig. 6A). Clustering analysis further revealed distinct divergences emerging among transcriptome profiles of all tested varieties under varying salinity levels (Supplementary Fig. 6B).

**Figure 3:**
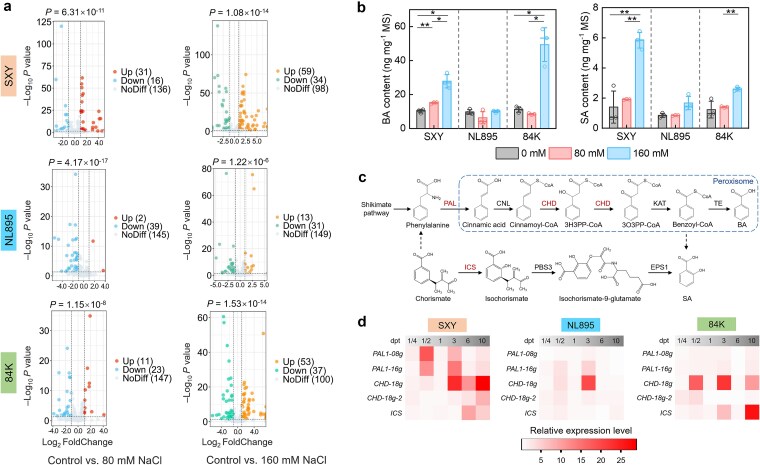
Salt-responsive phenolic acid exudation from poplar roots. (**a**) Volcano plot of differentially expressed genes (DEGs) from phenylpropanoid biosynthesis pathway in poplar roots under salt stress. Up, number of the upregulated genes; down, number of the downregulated genes; NoDiff, number of genes with no difference in expression level in this comparison. The *P* value for enrichment of DEGs in the phenylpropanoid biosynthesis pathway in each genotype is calculated by hypergeometric test. (**b**) BA and SA content in root exudates of NaCl-treated poplar. Mean ± SD; n = 3 biologically independent samples; and different significance levels between treatments are marked with asterisks (^*^P < .05, ^**^P < .01; ANOVA, LSD). (**c**) Simplified BA and SA biosynthesis pathway. PAL1, phenylalnine ammonia lyase 1; CNL, cinnamate-CoA ligase. CHD, cinnamoyl-CoA hydratase/dehydrogenase; KAT, 3-ketoacyl-CoA thiolase. TE, thioesterase. BA2H, benzoic acid 2-hydroxylase. ICS, isochorismate synthase. PBS3, avrPphB SUSCEPTIBLE3. EPS1, ENHANCED PSEUDOMONAS SUSCEPTIBILITY 1. The reactions in the blue box are localized to the peroxisome. (**d**) Relative expression of BA and SA biosynthesis-related genes in roots of NaCl-treated poplar determined by qPCR. The x-axis of the clustering heatmap is colored according to normalized Z-scores, which represents the expression level of the corresponding gene.

To better understand the functions expressed in poplar roots in response to salt stress, we employed KEGG enrichment analysis and demonstrated that “phenylpropanoid biosynthesis” (pop00940) enriched the most in SXY whereas the lowest in NL895 (Supplementary Fig. 7). Volcano plot analysis further revealed that under low salinity, SXY exhibited the most upregulated and the fewest downregulated genes in this pathway (31 upregulated, 16 downregulated), whereas an opposite pattern was detected in NL895 (2 upregulated, 39 downregulated); under high salinity, the number of up-regulated genes was also associated with salt tolerance (59 in SXY, 53 in 84 K, and 13 in NL895), when similar quantities of down-regulated genes were observed across all genotypes (Fig. 3a). These results suggest that activation of the phenylpropanoid biosynthesis pathway under salt stress is positively correlated with salt tolerance in a dose-dependent manner. Subsequent qPCR targeting on 13 functional genes confirmed these results (Supplementary Fig. 8 and Supplementary Table 8). These results indicate that the salt-tolerant plants trigger phenylpropanoid biosynthesis pathway to respond to salt stress.

Phenylpropanoid biosynthesis is the main pathway to form phenolic acids that are common root exudates to orchestrate the plant-microbe crosstalk under stress [10, 43, 44]. Thus, we measured the levels of phenolic acids in root exudates of poplar varieties under salt stress through targeted metabolomic method, and found genotype-specific phenolic acid secretion patterns (Supplementary Fig. 9). Among all tested phenolic acids, BA levels increased by 47% and 86% in SXY under low and high salinity, respectively, compared to control, and SA accumulation reached 1.4-fold and 3.1-fold. For 84 K, root exudates contained 4.8-fold higher BA and 85% higher SA only under high salinity relative to control groups (Fig. 3b and Supplementary Fig. 10). These results indicate that BA and SA increased along with salinity in SXY, accumulated only under high salinity in 84 K, but did not alter in NL895.

We analyzed the expression of genes involved in BA and SA biosynthesis in poplar roots under salt stress (Fig. 3c and Supplementary Table 8). Among these genes, *cinnamoyl-CoA hydratase/dehydrogenase-18 g* (*CHD-18 g*; Podel.18G080700) showed the strongest induction. Its expression level in salt-stressed SXY peaked at 28-fold that of the control, whereas the induction reached 22-fold and 19-fold in 84 K and NL895, respectively (Fig. 3d and Supplementary Fig. 11). These results indicate that the activation level of *CHD-18 g* under salt stress showed a positive correlation with plant salt tolerance. Overall, salt-tolerant plants upregulate *CHD-18 g* and secrete phenolic acids, predominantly BA and SA, into the rhizosphere in response to salt stress.

### BA promotes the growth of *Pseudomonas stutzeri*

**Figure 4:**
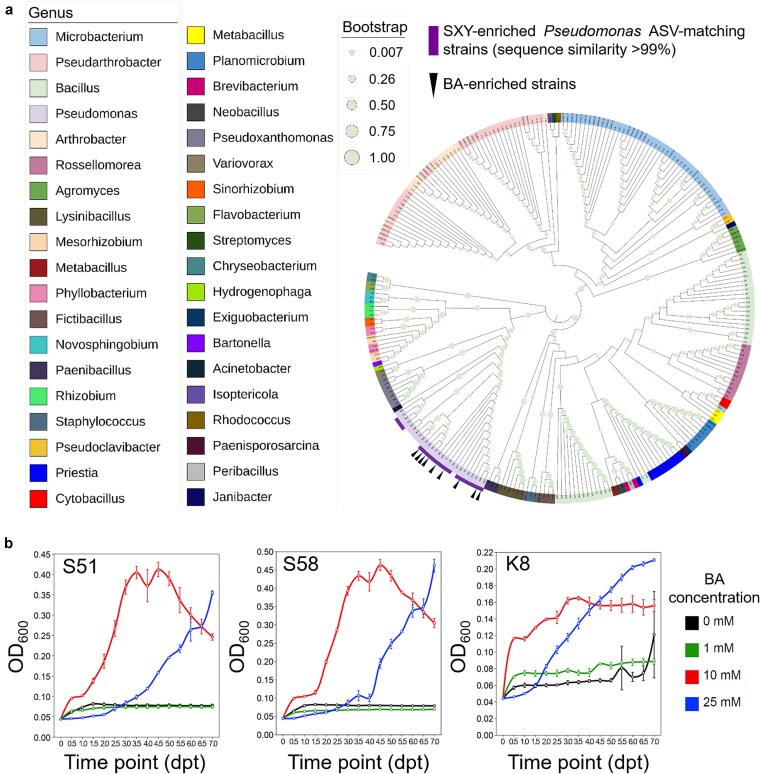
The phenolic acid-induced *Pseudomonas* taxa. (**a**) Phylogeny of the bacterial strains isolated from the poplar rhizosphere under salt stress. The first outer ring with black triangles represents BA-induced strains. The second outer ring with purple bars stands for the isolated strains that match the salt-enriched rhizosphere *Pseudomonas* ASVs (sequence similarity >99%). (**b**) Growth curves of pseudomonads using BA as resource. Mean ± SD; n = 3 technical replicates.

To obtain in vitro evidence that phenolic acids directly enrich *Pseudomonas*, we performed an isolation campaign from poplar rhizosphere under salt stress. This effort yielded a total of 364 isolates including 19 *Pseudomonas* strains that share >99% sequence similarity to the SXY-enriched ASVs (Fig. 4a and Supplementary Table 9). We subsequently identified three highly responsive *Pseudomonas* strains, S51, S58, and K8, that showed >3-fold induction by BA in pure-culture experiments (Supplementary Fig. 12). They were phylogenetically classified as *P. stutzeri* (Supplementary Fig. 13).

To pinpoint whether these three isolates exploit BA as resource for proliferation, BA was used as a replacement for the original carbon source (soya peptone) during bacterial culture. We observed that OD_600_ of S51 and S58 were both elevated to almost 6-fold by 10 mM BA at 4.5 dpt compared to control (0 mM BA), but that of K8 only increased to 2.6-fold; at 7 dpt, treatment with 25 mM BA resulted in 4-fold and 5-fold increases in OD_600_ for S51 and S58, respectively, whereas K8 exhibited only a 100% increase compared with control (Fig. 4b). Moreover, this reaction was accompanied by the characteristic formation of brown compounds (Supplementary Fig. 14). Thus, BA promoted growth of all tested strains, particularly S51 and S58. These results provide direct evidence that BA can induce the proliferation of specific *P. stutzeri* taxa.

### *CHD-18 g*-dependent phenolic acid biosynthesis is involved in rhizosphere *Pseudomonas* enrichment

**Figure 5:**
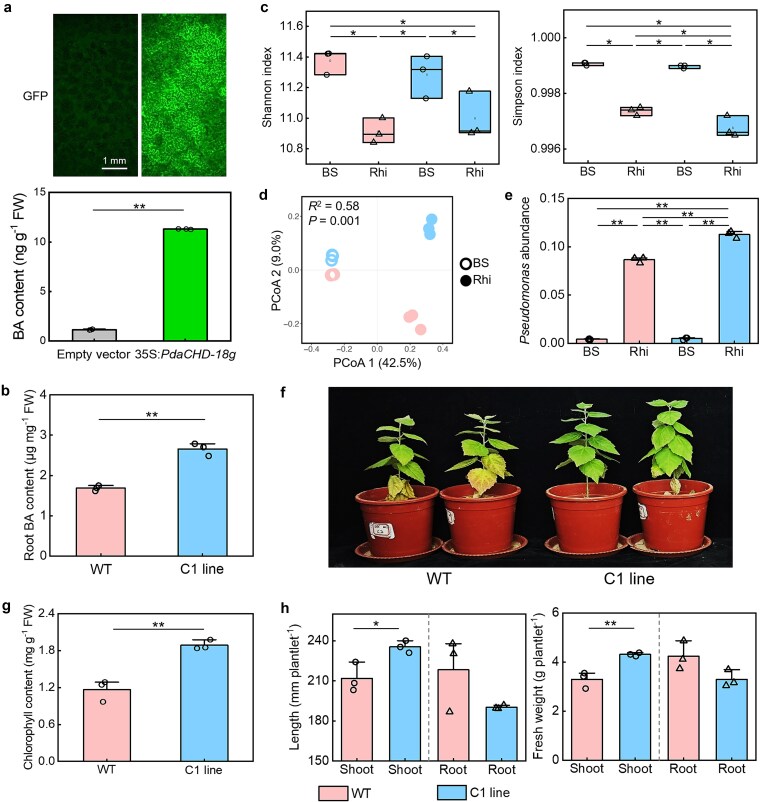
*CHD-18 g*-dependent phenolic acid biosynthesis and rhizosphere *Pseudomonas* enrichment. (**a**) BA level in tobacco leaves transiently expressing *PdaCHD-18 g*. Empty vector was used as control with green fluorescence protein (GFP) as tag. (**b**) Root BA levels of WT and SXY overexpressing *PdaCHD-18 g* (C1 line). (**c**) α-diversity, (**d**) PCoA for rhizobacterial community (PERMANOVA), and (**e**) relative abundance of rhizosphere *Pseudomonas* of WT and the C1 line under salt stress. **f**, Phenotypes, **g**, chlorophyll content, and **h**, biomasses of WT and the C1 line under salt stress. Mean ± SD; **abceg**, n = 3 biologically independent samples; **h**, n = 3 plantlets. Different significance levels between treatments are marked with asterisks (^*^P < .05, ^**^P < .01; ANOVA, LSD for **abceg**, and Student’s t test for **h**).

To explore the molecular regulation of root metabolism controlling the rhizosphere microbiome and its role in plant salt tolerance, we validated function of the salt-induced *CHD-18 g*. A previously characterized *CHD-18 g* ortholog in *Populus trichocarpa* (Potri.018G082900, exhibiting 99.9% sequence identity with Podel.018G080700; Supplementary Fig. 15) has been confirmed to mediate BA biosynthesis in peroxisomes [45]. Thus, we presumed that the SXY-derived *CHD-18 g*, designated *PdaCHD-18 g*, shares similar properties with its *P. trichocarpa* ortholog. We further performed transient expression of *GFP*-tagged *PdaCHD-18 g* in tobacco leaves to test this hypothesis (Supplementary Fig. 16A and Supplementary Table 8), and demonstrated that over-expression of *PdaCHD-18 g* resulted in a 10-fold increase of endogenous BA (Fig. 5a and Supplementary Fig. 16B). These results demonstrate that *PdaCHD-18 g* mediates phenolic acid biosynthesis in plant.

To examine the *in vivo* function of *PdaCHD-18 g* under salt stress, we generated transgenic poplar plantlets overexpressing *PdaCHD-18 g* (Supplementary Fig. 17 and Supplementary Table 8). This experimental strategy was adopted considering the existence of multiple CHD-encoding genes in poplar (e.g. Podel.018G080700 and Podel.18G082300). Knocking out one of them alone might trigger metabolic compensatory mechanisms obscuring the true phenotype. HPLC analysis revealed that the 35S:*PdaCHD-18 g* C1 line (C1 line for short) accumulated 58% more root BA than wild type (WT; Fig. 5b). We therefore chose the C1 line for subsequent experiments. High-throughput sequencing of the rhizosphere microbial community showed a decreased Simpson index in the C1 line compared to WT under salt stress (PERMANOVA for Fig. 5d; *R*^2^ = 0.58, *P* = .001; Fig. 5c and d, and Supplementary Table 10), suggesting that the transgenic plantlets assembled a more uneven rhizosphere bacterial community. Moreover, overexpression of *PdaCHD-18 g* increased the relative abundance of rhizosphere *Pseudomonas* by one third (from 8.7% to 11.3%; Fig. 5e and Supplementary Table 11). Phenotypic validation demonstrated that the C1 line performed better than WT under salt stress, exhibiting a 62% higher chlorophyll level, an 11% increase in shoot length, and a 31% elevation of shoot fresh weight (Fig. 5f to h). Thus, overexpression of *PdaCHD-18 g* enhanced salt tolerance and plant growth under salt stress. Taken together, these results suggest the existence of a *CHD-18 g*-dependent phenolic acid biosynthesis pathway that is implicated in the rhizosphere *Pseudomonas* enrichment and host salt tolerance.

### Phenolic acid-induced *Pseudomonas* support poplar against salt stress

**Figure 6:**
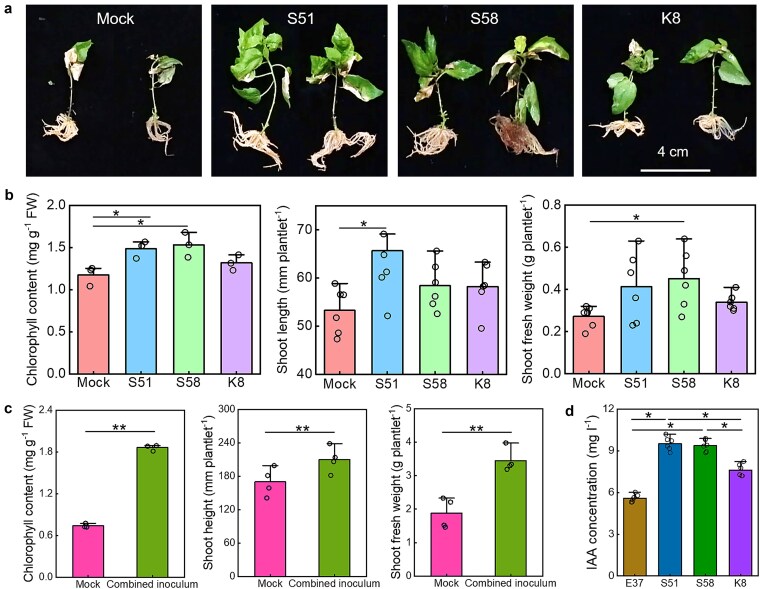
Phenolic acid-induced *Pseudomonas*-mediated plant salt tolerance. Effects of *Pseudomonas* strains on (**a**) phenotype, and (**b**) chlorophyll content and shoot biomass of SXY under salt stress. Mean ± SD; in chlorophyll content determination, n = 3 biologically independent samples; and in biomass measurement, n = 6 plantlets. Effects of the combined inoculum on (**c**) chlorophyll content and shoot biomass of SXY under salt stress. Mean ± SD; in chlorophyll content determination, n = 3 biologically independent samples; and in biomass measurement, n = 4 plantlets. (**d**) Indoleacetic acid (IAA) producing properties of *Pseudomonas* strains. Mean ± SD; n = 6 technical replicates. (**bcd**) Different significance levels between treatments are marked with asterisks (^*^P < .05, ^**^P < .01; ANOVA, LSD).

To examine the role of the phenolic acid-induced *Pseudomonas* taxa in the establishment of plant salt tolerance, we inoculated S51, S58, and K8 individually on the roots of NaCl-challenged SXY (Supplementary Fig. 18). These binary interaction assays demonstrated that all tested isolates alleviated salt-induced phytotoxicity and promoted plant growth compared to mock (Fig. 6a). In detail, inoculation with S51 had the most pronounced effect on host salt tolerance, evident from a 26% increase in chlorophyll content, a 23% increase in shoot length, and 17% and 80% increases in root length and fresh weight, respectively. The growth-promoting effect of S58 was reflected by a 30% increase in chlorophyll content and a 66% increase in shoot fresh weight. Inoculation with K8 improved plant performance under salt stress, but did not alter the measured growth parameters (Fig. 6b and Supplementary Fig. 19A). This provides clue that S51 showed a stronger effect than S58 and K8. These findings demonstrate that phenolic acid-induced *Pseudomonas* taxa improve poplar resilience to salt stress.

Considering the natural coexistence of microbes in the field, we tried to mimic natural condition by inoculating a combined inoculum comprising of S51, S58, and K8 to poplar rhizosphere. Consistent with expectations, the combined inoculum effectively restored salt tolerance and stimulated poplar growth under salt stress (Fig. 6c). Specifically, the combined inoculum led to a stronger salt-tolerant phenotype compared with mock, accompanied by a 1.5-fold increase in chlorophyll content, as well as 23% and 84% increases in shoot height and fresh weight (Supplementary Fig. 19B and C). These findings demonstrate that not only phenolic acid-induced *Pseudomonas* taxa but also their combined inoculum improve poplar resilience to salt stress.

To better understand the beneficial properties that *Pseudomonas* taxa employ to enhance plant salt tolerance, we evaluated their capabilities of indoleacetic acid (IAA) biosynthesis. For the negative control, we employed *Peribacillus frigoritolerans* E37 (E37 for short) isolated from the endosphere of poplar, which possesses low abilities to produce IAA. All tested *Pseudomonas* strains produced more IAA than E37, wherein S51 produced the greatest amounts in the assay, followed by S58 and then K8 (Fig. 6d). This could explain the more protective effects of S51 on poplar than other strains under salt stress (Fig. 6a and b). Taken together, the phenolic acid-enriched *Pseudomonas* taxa improve the host salt tolerance potentially through producing IAA.

## Discussion

Plants have evolved diverse mechanisms to withstand environmental stress, including not only well-characterized endogenous responses but also, more recently recognized, microbiome-mediated resilience [5, 46]. In this study, we demonstrated that rhizosphere bacteria contributed to salt tolerance in poplar through parallel cultivation experiments conducted in both mixed substrate and natural saline soil. Previous studies had shown that root microbiota play an important role in plant tolerance to stress [7, 11, 47]. Plants assemble characteristic microbial communities to cope with salt stress through specific metabolism [11, 48]. Thus, variations in salt response in different poplar varieties serve as a critical factor in restructuring unique root microbiota [30, 49]. We observed divergent growth performance across poplar varieties under salt stress, with the most pronounced differences in roots, accompanied by distinct chemical phenotypes (e.g. root exudation profiles). These differential responses may be attributed to their phylogenetic divergences derived from distinct geographical distribution. Unlike *Aigeiros* species (NL895) from humid southern China, *Leuce* species (SXY and 84 K) are mainly distributed in northern China, where soil salinization is common under arid conditions [34, 35, 50]. We observed stronger phenotypic responses to bacterial inoculation in SXY and 84 K than in NL895, implying that the *Leuce* species tend to cooperate with soil microbes under salt stress. These specific responses likely reflect genomic variations shaped by long-term interaction with adverse environments [51]. Within sect. *Leuce*, the divergent responses observed in SXY and 84 K may be ascribed to the distinct genetic backgrounds of their parental lines [52]. These findings underline specific molecular responses in different poplar genotypes under salt stress.

To elucidate the molecular response of poplar varieties to salt stress, we performed transcriptome sequencing and revealed a substantial up-regulation of the phenylpropanoid biosynthesis pathway in salt-tolerant poplars upon salt stress. This pathway activation could alter the profiles of phenolic acids in root exudates [43, 44, 53]. By combining targeted metabolomic analysis and qPCR, we revealed that salt-enhanced secretion of characteristic phenolic acids (e.g. BA and SA) was accompanied with a marked up-regulation of *CHD-18 g* that encodes a cinnamoyl-CoA hydratase/dehydrogenase. Furthermore, conducting transient over-expression of *CHD-18 g* in tobacco leaves demonstrated that the *CHD-18 g* mediates formation of BA and SA, consistent with previous studies showing its catalyzing role in a peroxisome-specific β-oxidative pathway for phenolic acid biosynthesis [54–57]. Because poplar harbors multiple CHD-encoding genes (e.g. *CHD-18 g* and *CHD-18 g-2* in Fig. 3c), we shifted from single-knocking out to over-expression to avoid metabolic compensation. By transforming *CHD-18 g* into poplar, we validated that *CHD-18 g* governs BA and SA biosynthesis in roots, which further generates the increased secretion of these phenolic acids in poplar rhizosphere under salt stress. Thus, the activation of *CHD-18 g* with its mediation of phenolic acid biosynthesis in poplar following salt stress constitute a primary defensive behavior. This gene is also implicated in plant defense against insects and pathogens. *CHD-18 g* expression was significantly induced in poplar by herbivory and in tobacco by viral inoculation [45, 58]. Moreover, silencing of *CHD* homologs compromises SA accumulation induced by a pathogen-derived elicitor in tobacco leaves [58]. This evidence indicates a universal function of CHD in plant stress resistance. Our data demonstrated that *CHD-18 g* exhibited a far stronger response to salt stress in roots (over 18-fold up-regulation across all tested varieties; Supplementary Fig. 11) than to herbivory or viral inoculation in leaves, where expression increased by no more than 5-fold. These inconsistent expression patterns may be attributed to differences in stress types and/or sampling tissues. The promoter of *Populus CHD-18 g* may contain more abiotic stress-responsive *cis-*acting elements [59–61]. Besides, roots may be more sensitive to environmental stress than leaves, especially to stresses originating from soil [62, 63]. Our findings provide a theoretical basis for genetic modification to sustain plant stress tolerance in agroforestry practice.

Metabolites, such as flavones, phytohormones, carbohydrates, and amino acids, can mediate plant-microbe communication under stress [26, 30, 64]. We unveiled that *CHD-18 g*-driven phenolic acid biosynthesis enhances plant salt tolerance through sculpturing root microbiota. Under aseptic conditions, salt-tolerant poplar plants reinforce the expression of *CHD-18 g* to augment the root exudation of BA and SA in response to salt stress. Previous studies have shown that various phenolic acids, such as 4-hydroxybenzoic acid, ferulic acid, 4-hydroxycinnamic acid, and derivatives of coumarins and cinnamic acid, were exudated in large quantities when plants were challenged by salt stress [65, 66]. However, although we identified several salt-induced genes associated with phenolic acid biosynthesis (Supplementary Fig. 8), the levels of most phenolic acids in root exudates exhibited no clear correlation with either salinity or plant salt tolerance, except for BA and SA (Supplementary Fig. 9). BA and SA not only play important roles in plant endogenous responses to salt tolerance (e.g. antioxidation and signal transduction) [67–70], but also serve as common and pivotal mediators modulating the assembly of root microbial communities [64, 71–73]. Moreover, they share a large portion of their biosynthetic pathway [56]. This suggests that poplar may have evolved a specific and efficient mechanism to selectively synthesize BA and SA via the peroxisomal β-oxidative pathway instead of other phenolic acids. By releasing these compounds into the rhizosphere, poplar can further modulate its root-associated microbiota. Thus, we performed pure-culture assays of BA with key rhizosphere microbes, mainly *Pseudomonas* taxa, and found that phenolic acids directly promote the proliferation of these microbial populations. This is consistent with existing reports that verify the BA-utilizing capability of *Pseudomonas* taxa [74, 75]. This evidence explains the preferential enrichment of *Pseudomonas* in the rhizosphere of salt-tolerant poplars. Correspondingly, pot experiments with *CHD-18 g*-overexpressing transgenic poplars exhibited elevated abundances of both root phenolic acids and rhizosphere *Pseudomonas* compared to WT. Thus, the interactions between phenolic acids and key microbes in the rhizosphere play a crucial role in establishment of plant salt tolerance. These findings advance our understanding of the molecular and ecological mechanisms governing plant stress tolerance.

A combined inoculum composed of phenolic acid-enriched *Pseudomonas* isolates enhanced plant resilience, raising questions about interactions among these strains and the role of these interactions in microbiome reorganization. Single-strain applications often fail in field due to interference from resident microbiota [76]. Thus, research has shifted toward synthetic microbial communities (SynComs) that are simplified natural consortia that retain core functions [77]. SynComs are typically designed according to microbe–microbe interactions [78], and can either directly promote plant growth or steer assembly of indigenous microbial communities to amplify rhizosphere functions [48, 79]. In our study, the combined inoculum improved chlorophyll content and shoot biomass more effectively than individual strains (Fig. 6b and c), suggesting synergistic interactions. Our findings provide candidate strains for constructing SynComs to improve tree adaptation for salt stress, which would inspire approach innovation for microbial application in agroforestry.

Although robust correlations were established between the candidate gene, root phenolic acid metabolism, and key microbial taxa, their causal relationships remain inferential. For instance, the role of key microbes was tested in parallel systems, lacking cross validation between poplar varieties. Though the candidate strains produce abundant IAA, how protective microbiome assemblages form remains largely unexplored. To obtain more direct evidence, future research will employ reciprocal inoculation experiments to confirm the protective function of rhizosphere microbes. Genome sequencing and loss-of-function assays will be combined to elucidate the molecular mechanism by which key microbes mitigate salt stress. Additionally, the role of SynComs in rhizosphere microbial assembly and synergistic functions need further clarification.

**Figure 7:**
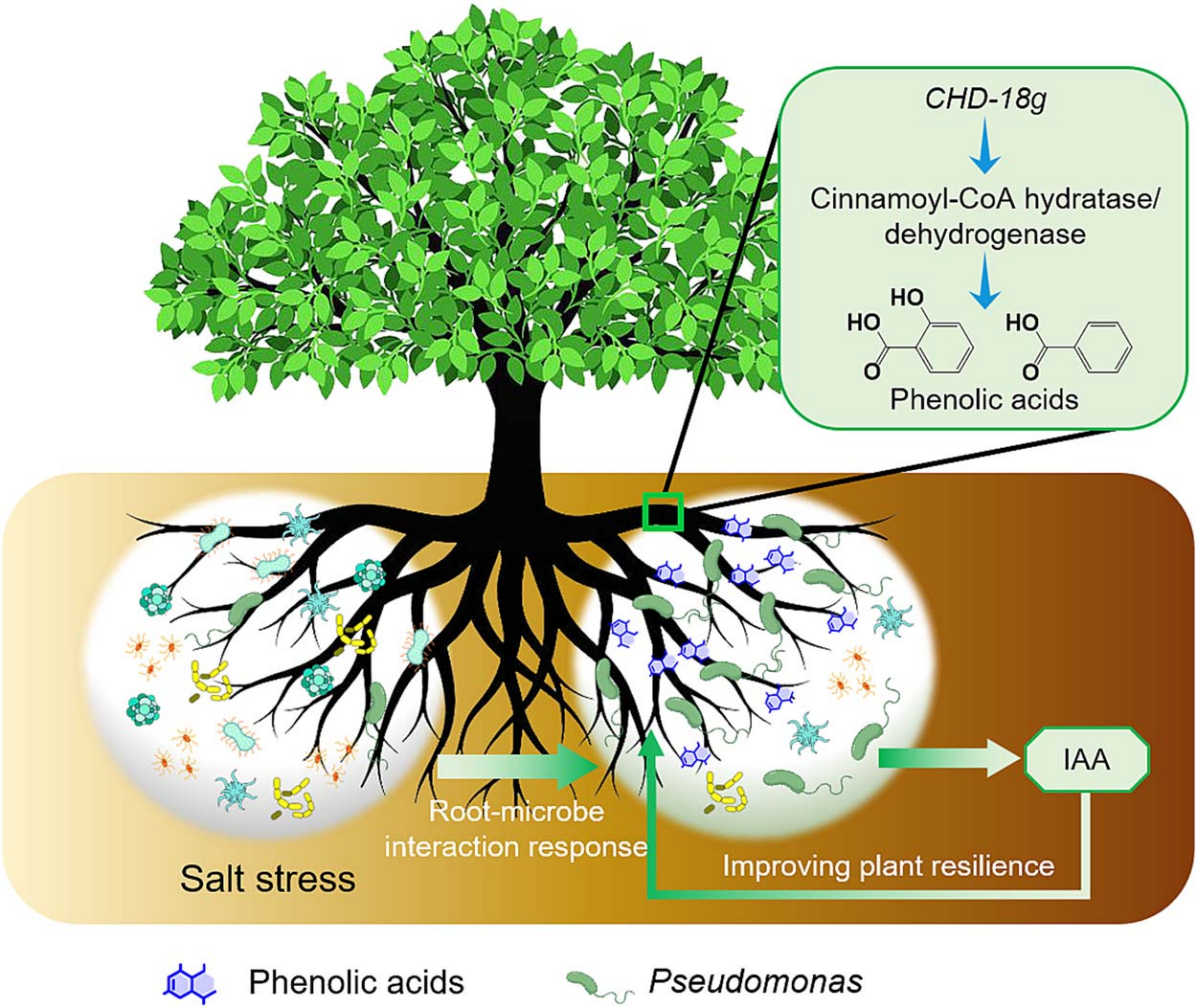
Proposed model of *CHD-18 g*-modulated rhizosphere *Pseudomonas* in shaping tree salt tolerance based on this work. Under salt stress, the salt-tolerant poplar variety triggers phenolic acids biosynthesis by activating *CHD-18 g* expression, and secrete them to recruit rhizosphere *Pseudomonas* from soil, helping the host endure salinity via IAA biosynthesis.

In conclusion, our study demonstrates that genotype-dependent root metabolic responses are pivotal for establishing beneficial plant-microbiome interactions under salt stress. Poplar roots recruit specific *Pseudomonas* taxa via salt-induced exudation of phenolic acids, a process dependent on host capacity to activate *CHD-18 g*-mediated biosynthesis (Fig. 7). Based on these findings, implementing genetic modification to the key candidate gene, or pairing specific root metabolites as prebiotics with functional microbes to establish synbiotics would facilitate microbial application in agroforestry practice.

## Supplementary Material

Supplementary_material_wrag138

## Data Availability

All raw plant RNA-seq data and rhizosphere bacterial 16S rRNA sequencing data reported in this paper were deposited in the Sequence Read Archive (http://www.ncbi.nlm.nih.gov/sra). Raw 16S rRNA gene amplicon sequences derived from microbial inoculation experiments are publicly available under NCBI BioProject number PRJNA1305648. All 16S rRNA gene sequence data derived from natural soil culture experiments are publicly available under NCBI BioProject number PRJNA1305643. Raw 16S rRNA gene amplicon sequences derived from transgenic poplar plants are publicly available under NCBI BioProject number PRJNA1305630. The RNA-seq data for poplar roots are publicly available under NCBI BioProject number PRJNA1305737.
