# Supplementary material for: CHD-18 g-modulated Pseudomonas taxa support poplar salt tolerance
Source: ISME J. 2026 May 28;20(1):wrag138. doi: 10.1093/ismejo/wrag138 (PMC13332713; doi:10.1093/ismejo/wrag138)
Supplement: Supplementary_material_wrag138 [file supplementary_material_wrag138.zip › SI Materials and Methods-no track 26.5.27.pdf]

Supplementary Information

Materials and Methods

MS medium, culture, and acclimation conditions for axenic poplar plantlets

Murashige and Skoog (MS) medium was supplemented with the same formula and autoclaved at 120°C for 20 min as described in our previous work [1, 2], including 6-benzylaminopurine (6-BA, 4.0 mg l^{-1}), naphthalene acetic acid (NAA, 0.4 mg l^{-1}), thidiazuron (TDZ, 0.04 mg l^{-1}), sucrose (25 g l^{-1}), and agar (5.5 g l^{-1}). For subculture, 0.2-mm shoot tips were treated according to the following steps. The tissues were transferred to 1/2 MS medium with 6-BA (4.0 mg l^{-1}), NAA (0.2 mg l^{-1}), sucrose (25 g l^{-1}), agar (5.5 g l^{-1}), and pH 5.3, and were grown into plantlets under a 16/8 h light/dark period, illumination intensity of 360 µmol m^{-2} s^{-1} and day/night temperatures of 23/23°C.

Prior to pot experiment, poplar plantlets were acclimated to air humidity for 12 h, transferred into a commercial nutrient substrate (Xiangzheng, Hunan, China) for 15 days, and watered once a week with sterile ddH_2O (autoclaved at 120°C for 20 min). This nutrient substrate contained peat, desalted coconut coir, perlite, and vermiculite was sterilized at 120°C for 40 min in advance. The RDN-1000G-3 growth chamber (Dongnan Instrument, Ningbo, China) maintained controlled condition including a 16/8 h light/dark period, day/night temperatures of 25/20°C, and a relative humidity of approximately 70%.

Bacterial suspension preparation

Bacterial suspension was exacted as previously described [3]. Saline soil (10 g) was co-incubated with 10 ml of sterile ddH_2O in a petri at 26°C for 7 days, to restore the bacterial activity. The soil sample with ddH_2O was then thoroughly transferred to a sterilized 250 ml conical flask autoclaved at 120°C for 40 min, and diluted to 100 ml using sterile ddH_2O. The conical flasks were shaken at 4°C, 200 rpm for 1 h. The suspension was then sonicated twice at 47 kHz for 1 min, and then shaken again for 0.5 h. Afterwards, fungi in the suspensions were filtered out using a 10 µm filter membrane.

Inoculation of bacterial suspension to the mixed substrate

The prepared bacterial suspension at a concentration of 10 ml kg^{-1} was uniformly nebulized onto surface of the autoclaved mixed substrate with a spray bottle sterilized with 75% (v/v) ethanol. Sterile ddH_2O was used as mock. Subsequently, the mixed substrate was thoroughly mixed using a shovel subjected to the same 75% (v/v) ethanol sterilization treatment.

Photography, biomass measurement, and response value calculation

Photographs were taken on the 30th and 60th day post treatment (dpt), with biomasses of shoot and root measured on the same day. The survival rate was calculated at 60

dpt. The experiments were independently performed four times. We calculated the response trait values as described in our previous study with minor modifications [4]:

Response (%) = $(Tb - Tc)/Tc \times 100$,

where *Tb* is the trait value in the treatment group (0.27% and 0.58% NaCl with bacterial suspension) and *Tc* is the trait value in the control group (the corresponding NaCl treatment without bacterial suspension). The differences in each trait between the treatment group and the control group within each poplar variety were compared using Analysis of variance (ANOVA) and Student's t test.

DNA extraction for soil and mixed substrate and 16S rRNA gene sequencing

Total DNA was extracted from 0.3 g samples of bulk and rhizosphere soil or mixed substrate using the Fast DNA spin kit for soil (MP Biomedicals, Santa Ana, CA, USA). The obtained DNA was quantified using a NanoDrop5000 spectrophotometer (Thermo Scientific, Wilmington, USA), and qualified by 1.2% agarose gel electrophoresis. The V3-V4 region of the 16S rRNA gene was amplified using the universal primers 338F (5'- ACTCCTACGGGAGGCAGCA-3')/806R (5'-GGACTACNNGGGTATCTAAT-3') [5]. After purification using Vazyme VAHTSTM DNA clean beads (Vazyme, Nanjing, China), sequencing libraries were prepared using TruSeq Nano DNA LT Library Prep Kit (Illumina Inc., San Diego, CA, USA).

For 16S rRNA gene sequencing, sequence denoising or ASV clustering were conducted following the analytical pipelines implemented in USEARCH v10.0 (http://www.drive5.com/usearch/download.html) [6] and the DADA2 workflow within QIIME 2 (https://docs.qiime2.org/2019.7/tutorials/overview/) [7]. The sequencing depth ranged from 22238 to 97385 reads. Low-quality reads were discarded and paired sequences were merged. ASV was generated via the DADA2 pipeline and annotated using the Silva database (SILVA 138.2 at https://www.arb-silva.de/). The sequence of all samples was rarefied to a uniform sequencing depth prior to downstream bioinformatic analyses. The ASV table for each sample were compiled and summarized across all hierarchical levels of taxonomic classification.

Root mRNA extraction, **RNA sequencing, and transcriptomic analysis**

The total mRNA of root was extracted using the HiPure HP Plant RNA Mini Kit (Magen, Guangzhou, China) for mRNA-seq analysis following the manufacturer's instructions. mRNA was purified by the interaction of the poly (A) tails and magnetic oligo (dT) beads, and double stranded cDNA was synthesized to construct cDNA libraries for sequencing. The prepared cDNA libraries were examined by an Agilent High Sensitivity DNA Kit on an Agilent 2100 Bioanalyzer (Agilent, St. Clara, CA, USA) with an average fragment length of 200 to 300 bp. The libraries were sequenced with the Novaseq 6000-PE150 System (Illumina) to generate paired-end reads 150 bp in length.

Transcriptome was analyzed following the procedure of our previous report [1]. Raw data of FASTQ were generated by the sequencing platform. Connectors and low-quality Reads were filtered using Cutadapt (v1.16), and clean reads were used for mapping analysis with Bowtie2 (2.2.6) and Tophat2 (2.0.14), and the reference

genome for gene annotation was *Populus deltoides* WV94 V2.1 (https://phytozome-next.jgi.doe.gov/). HTSeq (0.9.1) and DESeq (1.30.0) were used for gene expression analysis at different expression levels. The *R* package edgeR was used to identify the differentially expressed genes (DEGs) [8]. FPKM was used to normalize the expression amount. DEGs were analysed by DESeq, and the conditions for screening differentially expressed genes were: $|\log2\text{FoldChange}| > 1$, $P < 0.05$. The threshold of the P value was determined using a false discovery rate in multiple tests. The PCA, KEGG enrichment, volcano plots and heatmaps were performed with tools from genescloud, a free online platform for data analysis (https://www.genescloud.cn).

qPCR cycling conditions and expression level calculation

The cycling conditions were as follows: 95°C for 5 min, followed by 40 cycles of denaturation at 95°C for 15 s, and annealing at 60°C for 30 s. Fluorescent signals were collected during the 60°C step. To validate the amplification of a single product, a melt curve, with conditions as 95°C for 15 s, 60°C for 1 min, and 95°C for 15 s, was generated at the end of the PCR cycles using software offered by the Step One Real-Time PCR Detection System (ThermoFisher Scientific, Waltham, MA, USA).

The threshold cycle (Ct) value of the target gene was normalized against that of the internal reference gene to yield a ΔCt value, which was further compared with the control group to calculate the ΔΔCt value. The fold changes in expression level relative to the control were expressed as $2^{-\Delta\Delta Ct}$. The *actin* gene was employed as the reference gene on account of its involvement in cell shape stabilization and its constitutive expression pattern in plant eukaryotic systems [9].

Pretreatment of MS medium for phenolic acids profiling

Medium samples treated with 2 ml of 4 M NaOH, and then hydrolyzed at 40°C for 2 h in a gas bath with shaking and protection from light, with pH adjusted to 2.0 by adding 4 M HCl. The mixture was shaken with 2 ml of n-hexane at room temperature for 20 min to remove the n-hexane layer, extracted the aqueous layer using ethyl acetate (2 × 2 ml), and then concentrated to nearly dry on a rotary evaporator at 35°C under reduced pressure. Before analysis, the residue was dissolved in 200 µl of 50% methanol/ddH_2O and transferred to insert-equipped vials. The extracts were then used for UPLC-MS.

Analytical conditions and the ESI source parameters for UPLC-MS assay

The analytical conditions were as follows, UPLC: column, Waters ACQUITY UPLC HSS T3 (1.7 µm, 2.1 mm × 50 mm; Waters Corporation, Milford, MA, USA); column temperature, 40°C; flow rate, 0.3 ml min^{-1}; injection volume, 2 µl; solvent system, water (0.1% acetic acid): acetonitrile (0.1% acetic acid) ; gradient program, 90:10 (v/v) at 0 min, 90:10 (v/v) at 2.0 min, 40:60 (v/v) at 6.0 min, 40:60 (v/v) at 8.0 min, 5:95 (v/v) at 8.1 min, 95:5 (v/v) at 12.0 min.

The mass spectrometry analysis data were recorded on a Q Exactive hybrid Q-Orbitrap mass spectrometer equipped with a heated ESI source (ThermoFisher Scientific) utilizing the selected ion monitoring MS acquisition methods. The ESI

source parameters were set as follows: spray voltage, −2.8 kV/3.0 kV; sheath gas pressure, 40 arb; aux gas pressure, 10 arb; sweep gas pressure, 0 arb; capillary temperature, 320°C; and aux gas heater temperature, 350°C. Data were acquired on the Q-Exactive using Xcalibur 4.1 (ThermoFisher Scientific), and processed using TraceFinder 4.1 (Clinical Research; ThermoFisher Scientific).

Rhizosphere bacterial isolation, purification, and sequencing

The rhizosphere compartment of poplar cultured in saline soil was suspended in PBS buffer, and then diluted to different concentrations (10^{-1} to 10^{-7}) and plated on tryptone soy agar (TSA, solid TSB; soya peptone 0.5%, NaCl 0.5%, casein peptone 1.5%, agar 1.5%, pH 7.3) at 26°C in the dark. After culture for 3 to 7 days, bacterial colonies exhibiting distinct colors, morphologies, and sizes were selected from the spread plates with sterilized inoculation loops. These isolates were subsequently re-streaked twice onto fresh TSA plates to obtain pure, single colonies [10]. There were total 364 colonies obtained. The 16S rRNA gene was amplified with primers 27F (5'-AGAGTTTGATCATGGCTCAG-3') and 1492R (5'-TACGGTTACCTTGTTACGACTT-3') of fresh colonies in replicate. PCR products were sequenced at Songon Biotech Co., Ltd. (Shanghai, China). All 16S rRNA gene sequences were submitted to NCBI databases for taxonomic identification.

Infiltration of tobacco leaves

Agrobacterium tumefaciens GV3101 expressing the *PdaCHD-18g* gene was grown on a yeast extract peptone (YEP) agar plate supplemented with 25 mg l^{-1} Rif and 50 mg l^{-1} Kan, cultured overnight in YEP with Kan and Rif at 28°C, 220 rpm and brought to an OD600 of 0.8 to 1.0, and then suspended in infiltration medium (1/2MS, 10 mM MES, pH 5.8, 10 mM $MgCl_2$, and 200 μM acetosyringone). After induction of virulence genes for 2 to 3 h in dark at room temperature, three fully expended leaves of 30- to 40-d stage *Nicotiana benthamiana* were infiltrated, with pCambia-1300-GFP empty vector as control. Leaf samples were collected 48 h later for BA level determination, and GFP was detected using a Carl Zeiss LSM 710 (Carl Zeiss Microscopy GmbH, Jena, Germany) inverted confocal laser-scanning microscope as described previously [11].

Genetic transformation of poplar

Transformation of poplar was performed as described in previous study [12]. GV3101 harboring *PdaCHD-18g* was employed for the genetic transformation to leaf discs of SXY. The strain carrying overexpression vector was inoculated into LB supplemented with 50 mg l^{-1} Kan and 50 mg l^{-1} rif, and shaked to an OD_{600} of 0.6 to 0.8. Subsequently, the Agrobacterium cells were harvested by centrifugation and resuspended in liquid 1/2 MS to prepare the infection suspension. Leaf discs of SXY were immersed in the Agrobacterium infection suspension for 10 to 15 min. Afterwards, the leaf discs were initially transferred to transformation medium containing MS with 0.4 mg l^{-1} 6-BA, 0.1 mg l^{-1} NAA, 0.01 mg l^{-1} TDZ, 100 mM acetosyringone, 300 mg l^{-1} tim, and 300 mg l^{-1} Cef. The addition of tim and Cef was

intended to suppress excessive proliferation of Agrobacterium. After 3 days of transformation, the leaf discs were transferred to screening medium (MS + 0.4 mg l^{-1} 6-BA, 0.1 mg l^{-1} NAA, 0.01 mg l^{-1} TDZ, 100 mM acetosyringone, 300 mg l^{-1} tim, 300 mg l^{-1} Cef, and 50 mg l^{-1} Kan) for further culture. The resistant buds were transplanted to rooting medium consisted of MS supplemented with 0.3 mg l^{-1} IBA, 50 mg l^{-1} Kan, and 300 mg l^{-1} Cef. The DNA of transgenic poplar was extracted by a plant genome extraction kit (Tsingke, Beijing, China), and positive lines were selected using primers in Supplementary Table S8.

Extraction of BA from plant tissues

Samples (0.1g) were treated with 2 ml of 0.3% NaOH (m/v), ultrasonicated at 70 Hz for 10 min, to homogenate, and then cool soaked for 10 min. Afterwards, the mixture was ultrasonicated for 20 min, and centrifuged at 13000 rpm for 20 min. The supernatant was mixed with 1 ml 0.3% $HClO_4$ (v/v), and pH was adjusted to 7.0. Next, the above solution was dried with nitrogen flow, and diluted with 0.5 ml ddH_2O. After filtration through a 0.22 μm membrane, the supernatant was ready for HPLC analysis.

Analytical conditions of HPLC for BA quantification

Column temperature, 35°C; flow rate, 1 ml min^{-1}; injection volume, 20 μl; and UV detection, 230 nm. Standard was used to quantify BA level, and the peak in different samples was calibrated according to integration of the peak area, to compare the content of BA between the control and treatments.

Chlorophyll content detection

Total chlorophyll of the poplar leaves was extracted by soaking the fresh leaf sample in 95% ethanol in darkness for 72 h. The absorbance of the extraction determined at 646 nm and 663 nm via a spectrophotometer (Beckman, Pasadena, CA, USA) was used to calculate the chlorophyll content. Leaves were pooled from at least three plantlets in each treatment and then homogenized. 0.3 g of leaves constitute one biological replicate. Three biological replicates were analyzed for each treatment, and the experiments were independently performed four times.

Bacterial IAA production determination

Bacterial isolates were cultured in TSB medium supplemented with 100 mg l^{-1} tryptophan. After incubation at 28°C, 180 rpm for 48 h, the bacterial cultures were centrifuged at 6500 rpm for 10 min to collect the supernatant. An aliquot of 1 ml supernatant was mixed with an equal volume of Salkowski's reagent, and the mixture was incubated at room temperature in the dark for 30 min. A pink coloration of the solution served as a qualitative indicator of IAA production. The reaction solution (200 μl) was pipetted into a 96-well microplate, and the absorbance was measured at 530 nm using a Cytation 5 microplate reader (BioTek, VT, USA). A standard IAA solution ranging from 10 to 50 mg l^{-1} was prepared, and a standard curve was constructed based on this concentration gradient. The IAA-producing capacity of each

bacterial strain was calculated quantitatively using the established standard curve.

Details of statistical analyses

Analysis of variance (ANOVA) was conducted, followed by Student's t test for plant biomass measurements, and the Least significant difference (LSD) test for most of the rest comparisons. Detailed information is provided in the corresponding figure legends.

Shannon and Simpson indices were calculated by using the "picante" and "vegan" *R* package, and PCoA was performed to ordinate the microbial composition in the different samples based on Bray-Curtis dissimilarity with the vegan and ggplot2 packages [1]. The differences among microbial communities were analyzed using Permutational Multivariate Analysis of Variance (PERMANOVA). Abundance data of genera were obtained using basic packages. All the above data were visualized with OriginPro (10.1.0.178). Linear discriminant analysis (LDA) Effect Size (LEfSe) analysis was performed using Wekemo Bioincloud (https://bioincloud.tech/). The heatmaps were generated using Heatmapper (http://www.heatmapper.ca/expression/). Phylogeny of the bacterial isolates was analyzed through the construction of a Neighbor-Joining tree utilizing MEGA 11, with a taxonomic cladogram generated by the iTOL web tool (https://itol.embl.de/) [13].

References

1. Liao YWK, Cao CY, Zhang QY. *et al*. Interaction between rhizobacterial community and host root determines poplar salt tolerance. *Land Degrad Dev* 2023; **34**:4415–4427. https://doi.org/10.1002/ldr.4786
2. Schmitz L, Yan Z, Schneijderberg M. *et al*. Synthetic bacterial community derived from a desert rhizosphere confers salt stress resilience to tomato in the presence of a soil microbiome. *ISME J* 2022; **16**:1907–1920. https://doi.org/10.1038/s41396-022-01238-3
3. Chen DL, Wang XX, Zhang W. *et al*. Persistent organic fertilization reinforces soil-borne disease suppressiveness of rhizosphere bacterial community. *Plant Soil* 2020; **452**:313–328. https://doi.org/10.1007/s11104-020-04576-3
4. Fu RX, Liu L, Wang ZC, *et al*. Synergy of diazotrophs with native soil microbes improves poplar traits. *Ind Crop Prod* 2025;**224**:120311. https://doi.org/10.1016/j.indcrop.2024.120311
5. Stringlis IA, Yu K, Feussner K. *et al*. MYB72-dependent coumarin exudation shapes root microbiome assembly to promote plant health. *Proc Natl Acad Sci USA* 2018;**115**:E5213–E5222. https://doi.org/10.1073/pnas.1722335115
6. Rognes T, Flouri T, Nichols B. *et al*. VSEARCH: a versatile open source tool for metagenomics. *PeerJ* 2016;**4**:e2584. https://doi.org/10.7717/peerj.2584
7. Bokulich NA, Kaehler BD, Rideout JR. *et al*. Optimizing taxonomic classification of marker-gene amplicon sequences with QIIME 2's q2-feature-classifier plugin. *Microbiome* 2018;**6**:90. https://doi.org/10.1186/s40168-018-0470-z
8. Robinson MD, McCarthy DJ, Smyth GK. edgeR: a Bioconductor package for differential expression analysis of digital gene expression data. *Bioinformatics*

2010; **26**: 139–140. https://doi.org/10.1093/bioinformatics/btp616
9. Yuan GQ, Gao HH, Yang T. Exploring the role of the plant actin cytoskeleton: from signaling to cellular functions. *Int J Mol Sci* 2023;**24**:15480. https://doi.org/10.1016/10.3390/ijms242015480
10. Zhou Y, Liu D, Li F. *et al.* Superiority of native soil core microbiomes in supporting plant growth. *Nat Commu* 2024;**15**:6599. https://doi.org/10.1038/s41467-024-50685-3
11. Chen MX, Hu TH, Xue Y. *et al*. *Arabidopsis* acyl-coenzyme-A-binding protein ACBP1 interacts with AREB1 and mediates salt and osmotic signaling in seed germination and seedling growth. *Environ Exp Bot* 2018; **156**:130–140. https://doi.org/10.1016/j.envexpbot.2018.09.007
12. Wang P, Zhou J, Sun WB. *et al*. Poplar CCR4-associated factor PtCAF1I is necessary for poplar development and defense response. *Int J Biol Macromol* 2023; **242**:125090. https://doi.org/10.1016/j.ijbiomac.2023.125090
13. Gu SH, Wei Z, Shao ZY. *et al.* Competition for iron drives phytopathogen control by natural rhizosphere microbiomes. *Nat Microbiol* 2020;**5**:1002–+. https://doi.org/10.1038/s41564-020-0719-8
