# Supplementary material for: CHD-18 g-modulated Pseudomonas taxa support poplar salt tolerance
Source: ISME J. 2026 May 28;20(1):wrag138. doi: 10.1093/ismejo/wrag138 (PMC13332713; doi:10.1093/ismejo/wrag138)
Supplement: Supplementary_material_wrag138 [file supplementary_material_wrag138.zip › Supplementary_Information-no_track-26.5.27_wrag138.pdf]

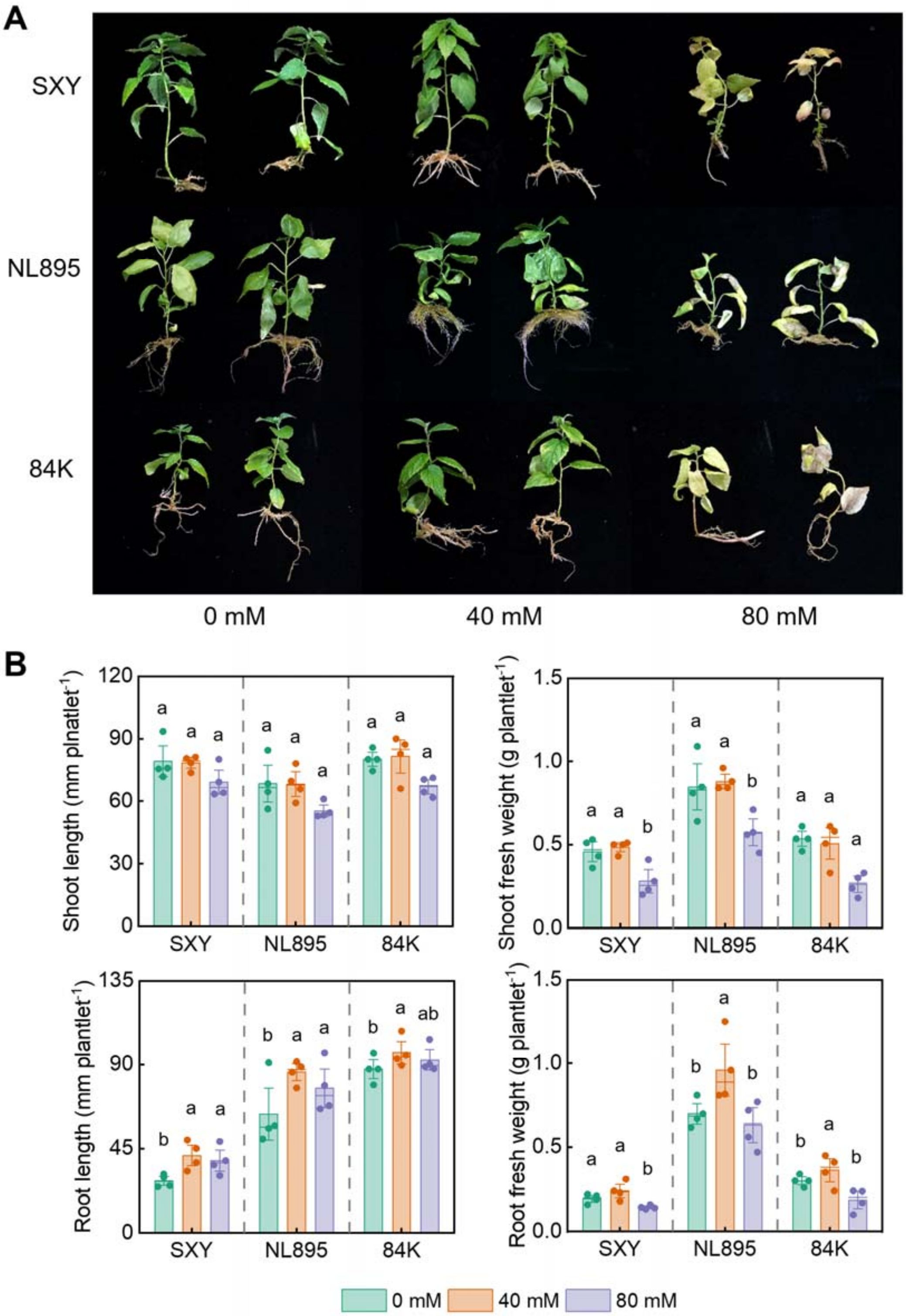

Supplementary Fig. 1 Salt tolerance of poplar varieties under sterile condition. **A**, Phenotypes of axenic poplar plantlets cultured in sterilized MS medium at 30 days post treatment (dpt). 0, 40, and 80 mM were NaCl concentration for treatments. **B,** Biomasses of plantlets in **A**. Mean ± SD; n = 4 biologically independent samples; and different letters indicate statistically significant differences among salt treatments within the same variety ($P < 0.05$; ANOVA, Duncan's test).

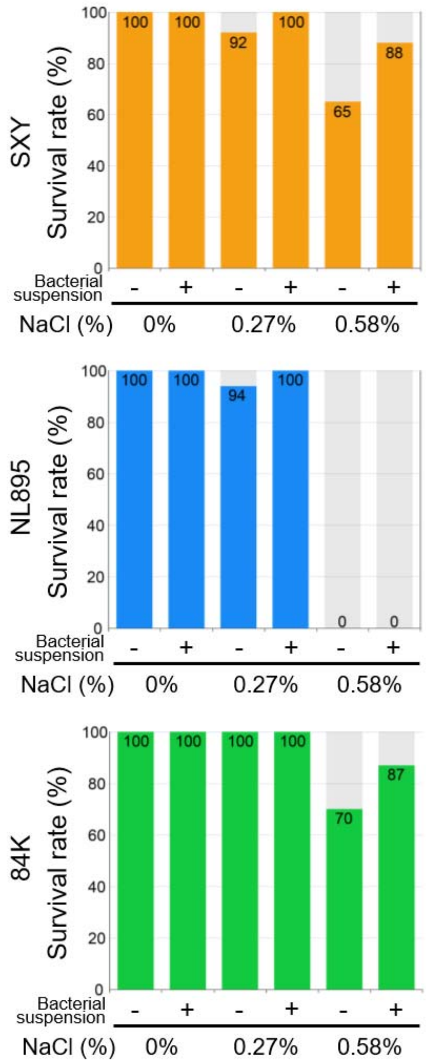

Supplementary Fig. 2 Effects of bacterial suspension on survival rate of poplar cultured in salt-treated sterile substrate. Data were recorded at 60 dpt.

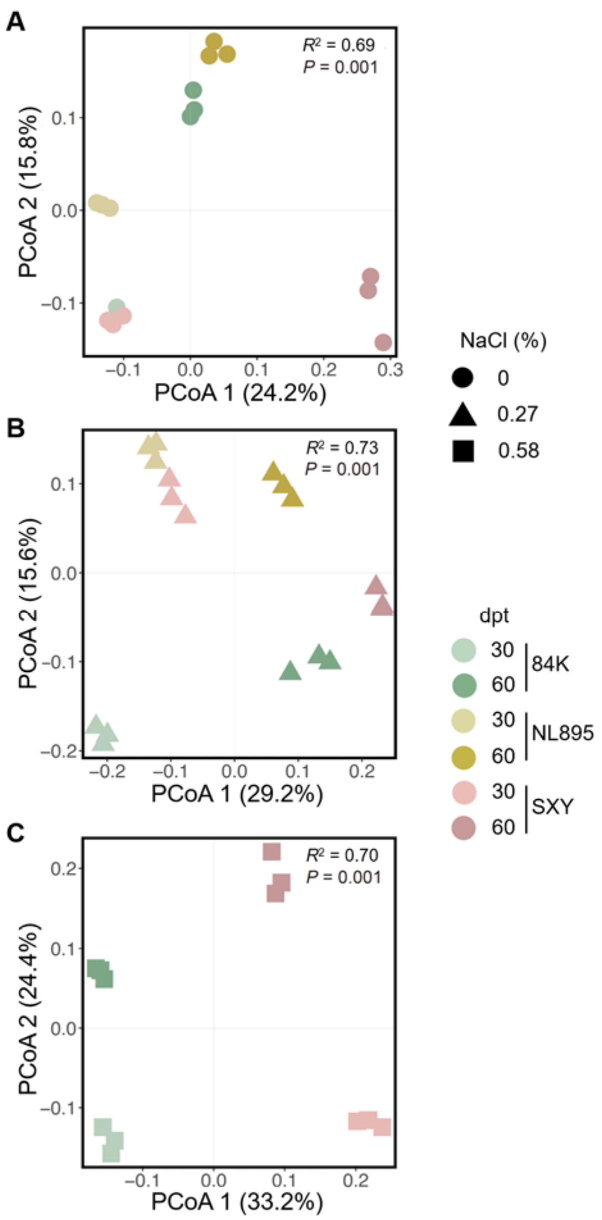

Supplementary Fig. 3 Principal coordinates analysis (PCoA) with Bray-Curtis dissimilarity metric performed across salinity levels in the bacterial inoculated soils. PCoA of rhizosphere bacterial community in soils from **A**, 0%, **B**, 0.27%, and **C**, 0.58% NaCl treatments under bacterial suspension inoculation at the ASV level. Statistical analysis is performed using permutational multivariate analysis of variance (PERMANOVA).

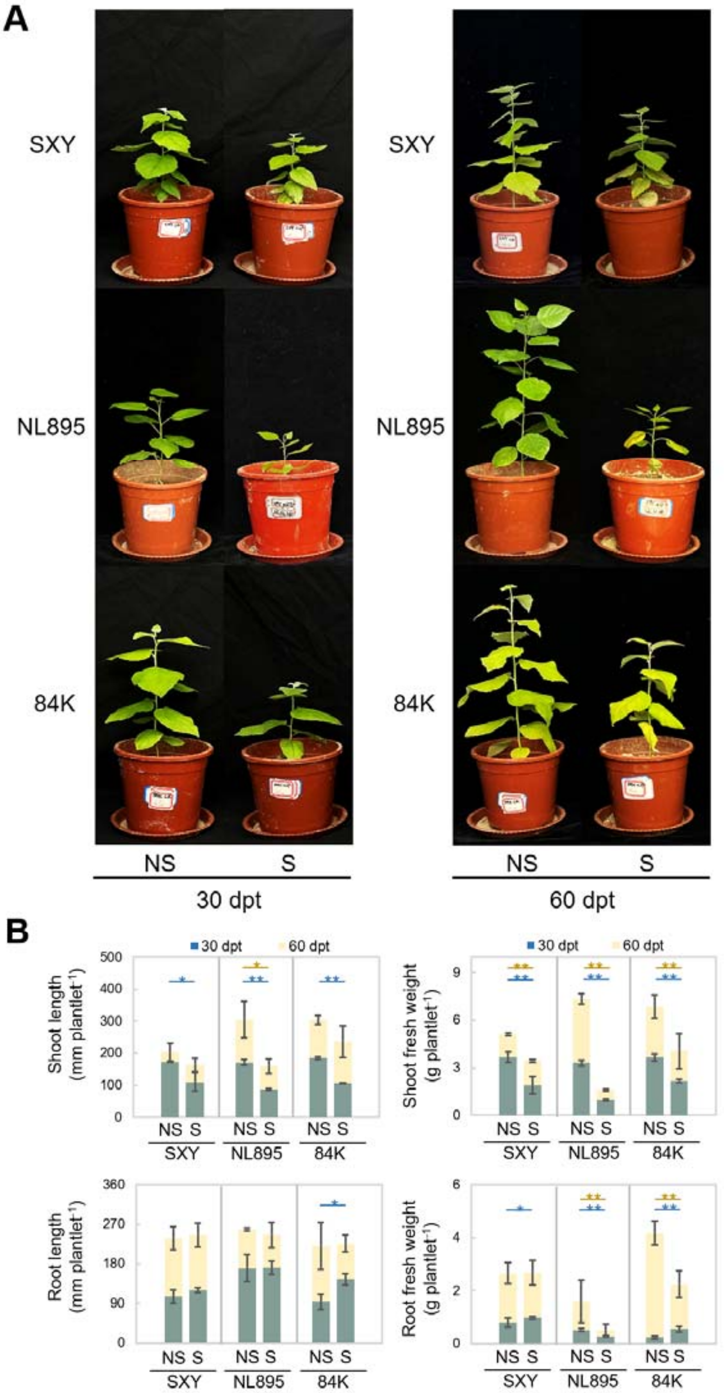

Supplementary Fig. 4 Salt tolerance of poplar cultured under non-sterile condition. **A**, Phenotypes and **B,** biomasses of poplar varieties cultured in natural soils. NS, non-saline soil (control); S, saline soil (salt stress). Blue asterisks represent significance of differences between treatments at 30 dpt, while the brown ones for 60 dpt. Mean ± SD; n = 3 plantlets; and significances were calculated between NS and S groups within the same variety (*P < 0.05, and **P < 0.01; ANOVA, paired student's t test).

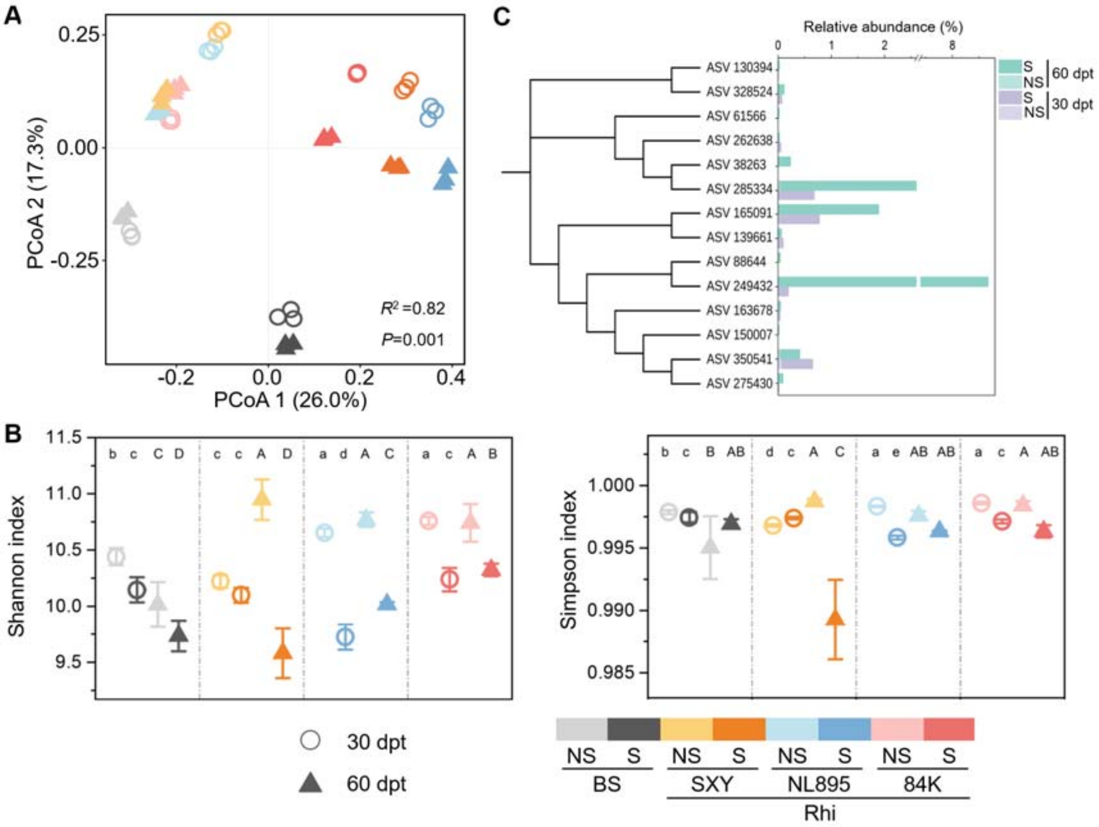

Supplementary Fig. 5 Difference in microbial community composition between poplar varieties cultured in natural soils. **A**, PCoA based on the Bray-Curtis dissimilarity (*P*=0.001, one-way PERMANOVA) and **B**, α-diversity for the bacterial community from bulk soil (BS) and the rhizosphere (Rhi) of poplar cultured in natural soils. Mean ± SD; n = 3 biologically independent samples. Different letters above the boxes indicate a significant difference (*P* < 0.05; ANOVA, Duncan's test). **C,** Neighbor-Joining tree of the SXY-enriched rhizosphere *Pseudomonas* ASVs and their relative abundance.

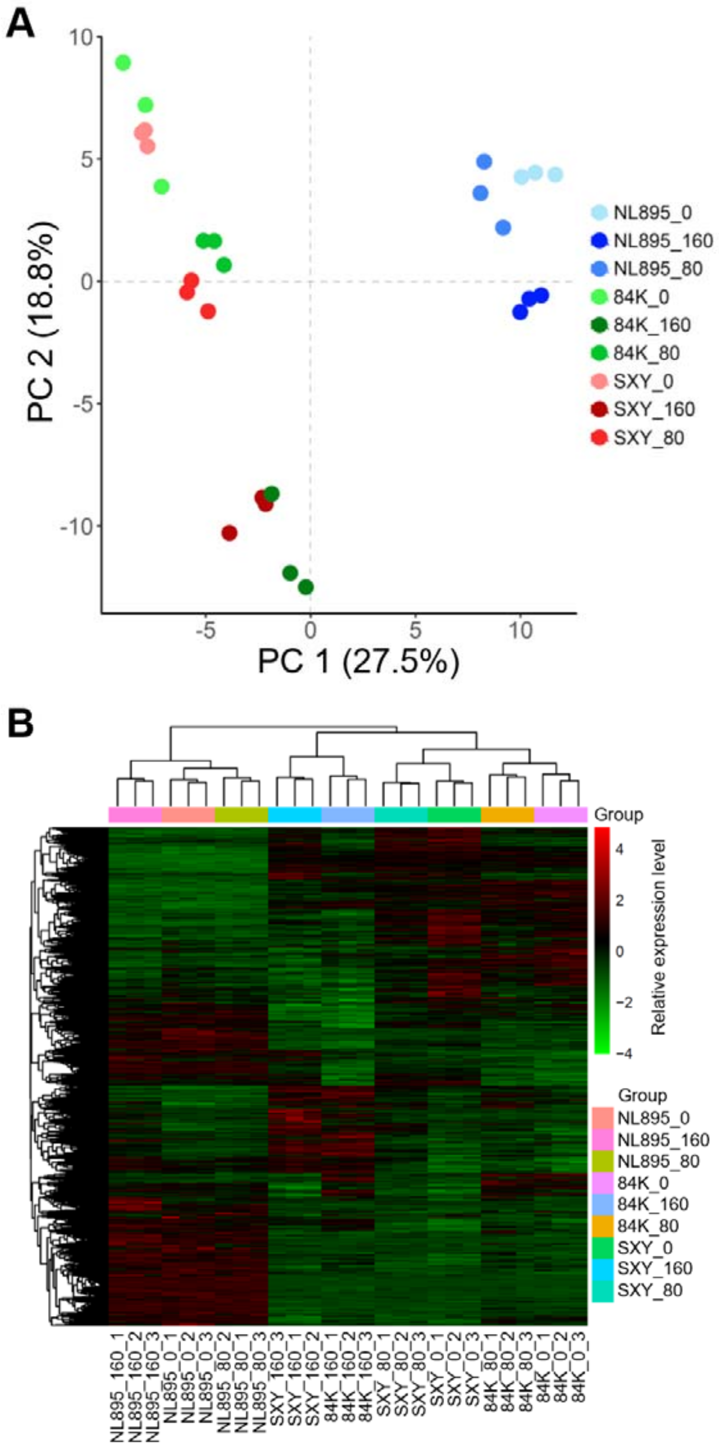

Supplementary Fig. 6 Transcriptomic diversity among the roots of poplar upon salt treatment. **A**, PCA showing the transcriptomic shift and **B**, the clustering analysis of DEG expression patterns in roots of poplar varieties. 0, 80, 160 indicate the dose of NaCl.

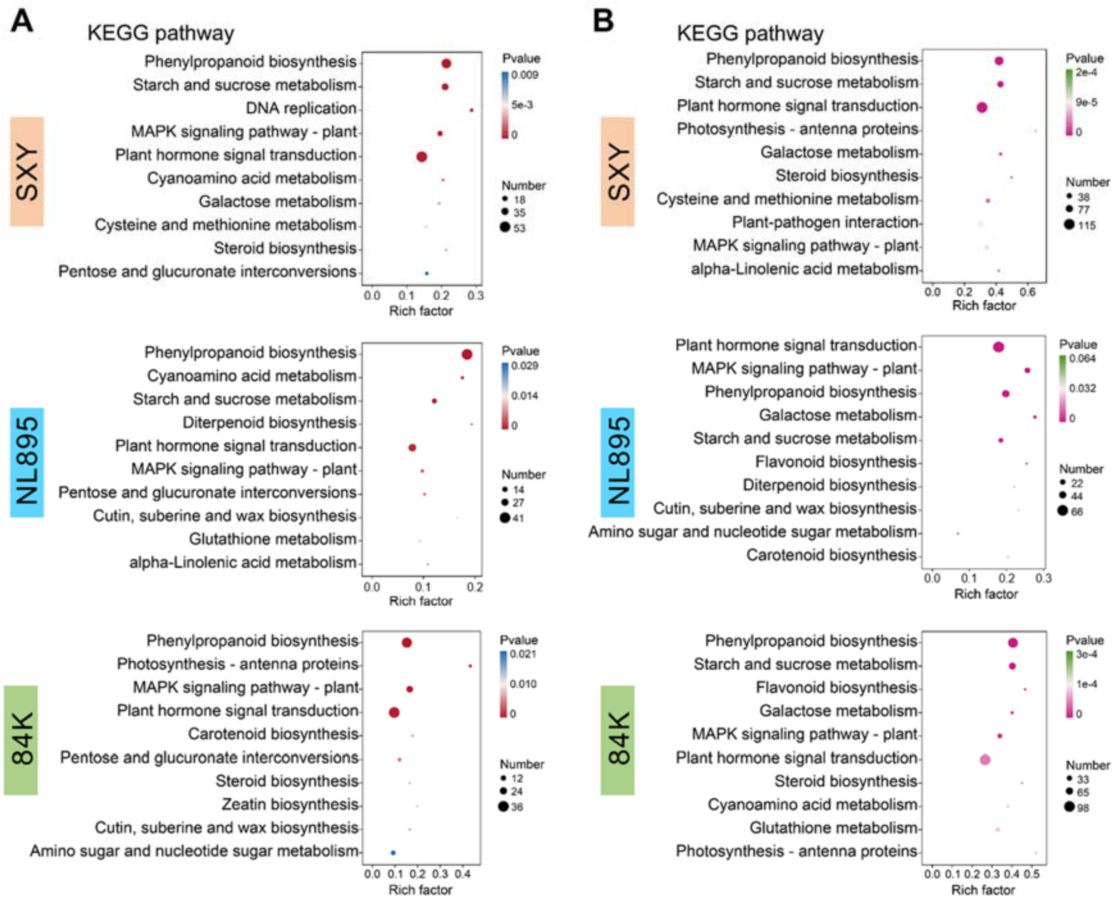

Supplementary Fig. 7 Illustration of top 10 enriched KEGG terms of transcriptome in poplar roots under salt stress. Each dot represents a KEGG term. The size of each dot indicates the number of genes associated with that KEGG term. The color scale of the dot represents the adjusted *P* value. **A**, Control vs. 80 mM NaCl; and **B**, control vs. 160 mM NaCl.

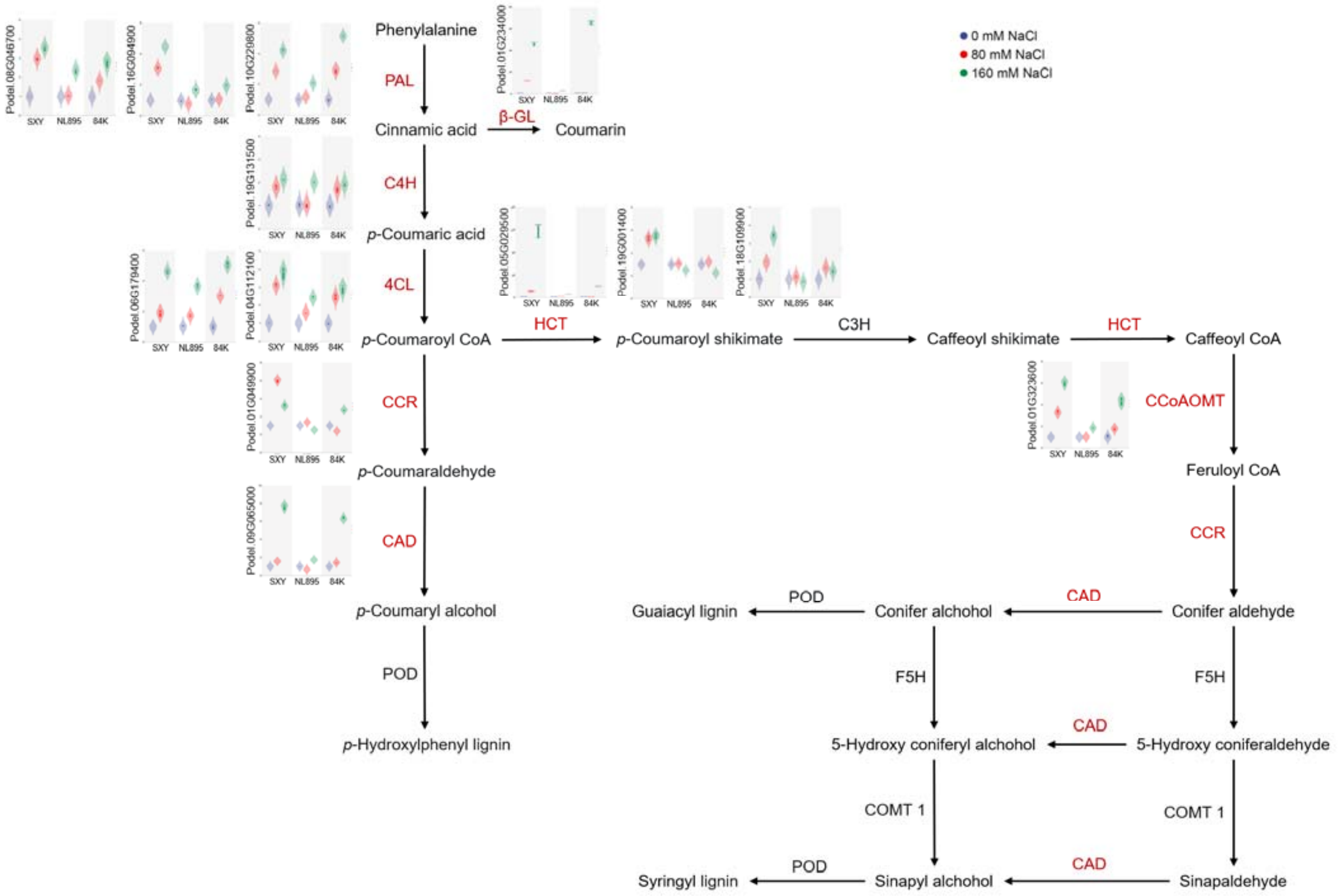

Supplementary Fig. 8 qPCR validation for the expression of genes involved in phenylpropanoid biosynthesis. PAL, phenylalnine ammonialyase; C4H, cinnamic acid 4-monooxygenase; 4CL, 4-coumarate:CoA ligase; HCT, hydroxycinnamoyl-CoA shikimate transferase; CCoAOMT, trans-caffeoyl-CoA 3-O-methyltransferase; CCR, cinnamoyl CoA reductase; CAD, cinnamoyl alcohol dehydrogenase; β-GL, β-glucosidase; C3H, p-coumarate 3-hydroxylase; F5H, ferulate 5-hydroxylase; COMT 1, caffeic acid O-methyltransferase 1; POD, peroxidase. Mean ± SD; n = 3 biological replicates.

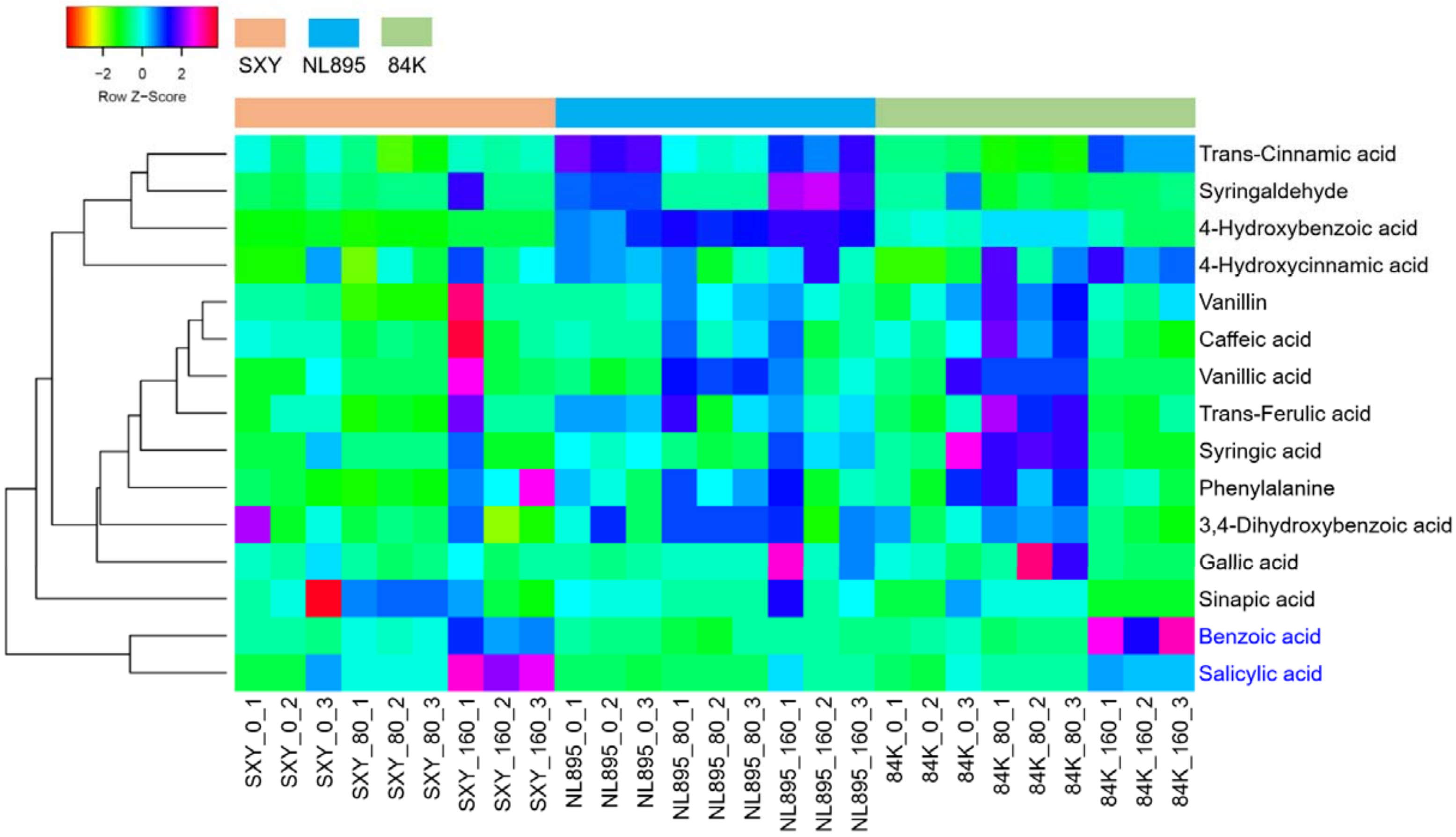

Supplementary Fig. 9 Response of phenolic acid profiles in root exudates of poplar to salt stress. 0, 0 mM NaCl; 80, 80 mM NaCl; 160, 160 mM NaCl. The x-axis denotes distinct salinity treatments applied to poplar varieties, and each clustering heatmap is color-coded based on normalized Z-scores calculated from content of the labeled phenolic acid. Each column represents one single biological replicate of the corresponding treatment.

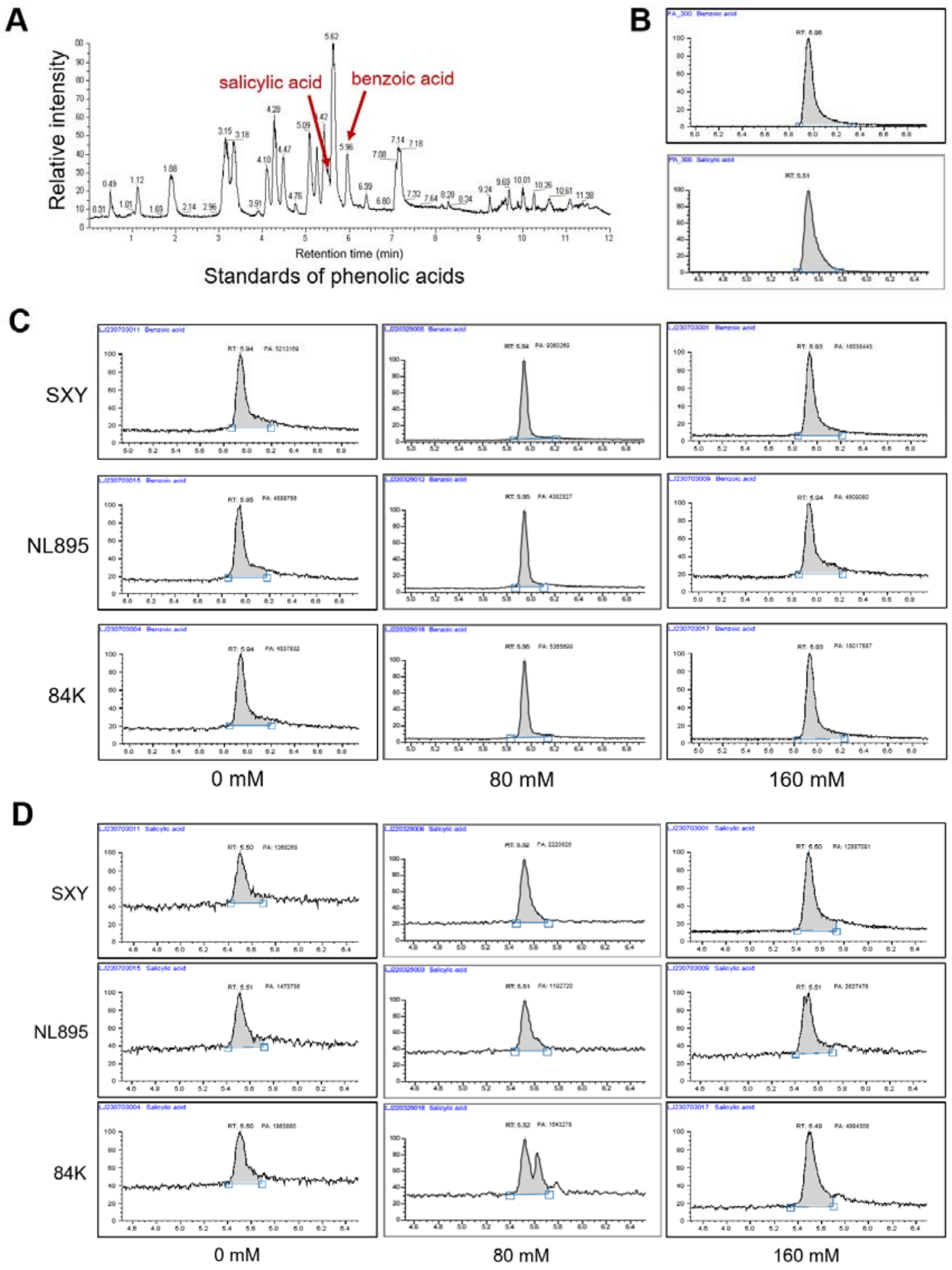

Supplementary Fig. 10 Determination of phenolic acids in root exudates of poplar. **A**, Ion flow of standards for phenolic acids quantified by UPLC-MS. **B**, Peak map for quality control samples of benzoic acid (BA, upper) and salicylic acid (SA, below). Peak map of **C**, BA and **D**, SA determination for root exudates of poplar varitetis. RT, Retention time; PA, peak area.

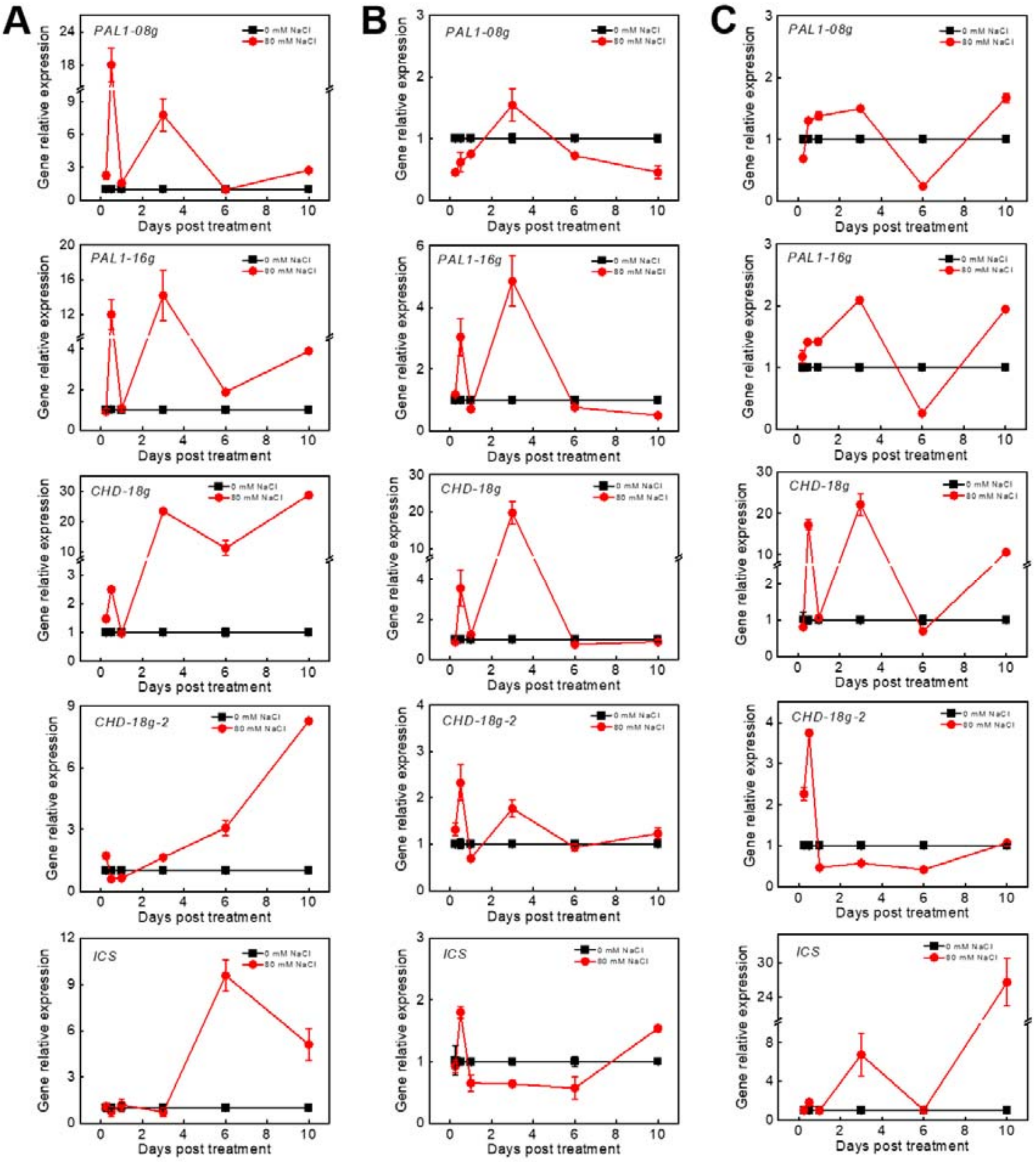

Supplementary Fig. 11 Relative expression of genes encoding enzymes involved in SA and BA biosynthesis. **A**, SXY. **B**, NL895. **C**, 84K. Black line, 0 mM NaCl (Control); red line, 80 mM NaCl (Salt). *PAL1*, *phenylalnine ammonia lyase 1*; *CHD*, *cinnamoyl-CoA hydratase/dehydrogenase*; *ICS*, *isochorismate synthase*. Mean ± SD; n = 3 biological replicates.

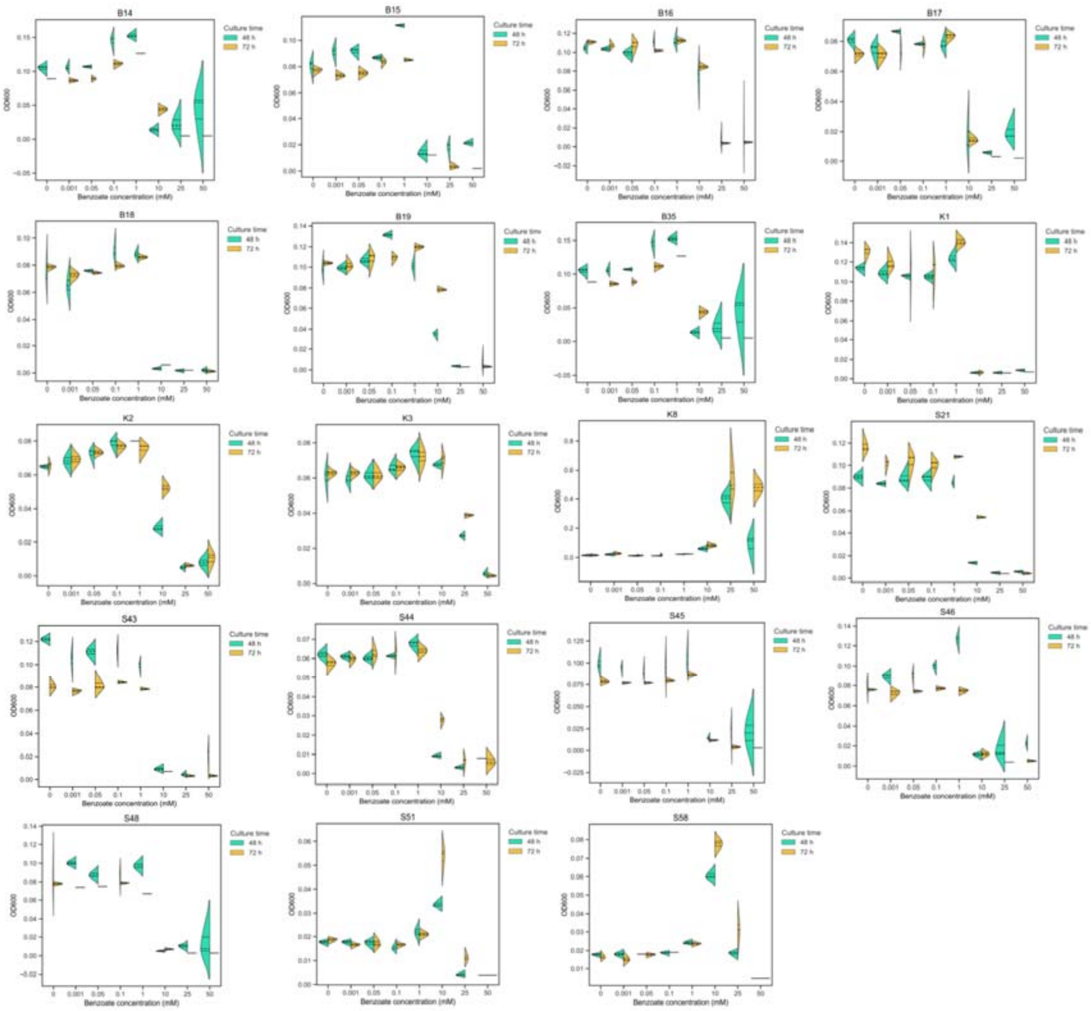

Supplementary Fig. 12 Effects of BA on the *Pseudomonas* isolates that match the SXY-enriched *Pseudomonas* ASVs. Mean ± SD; n = 3 technical replicates.

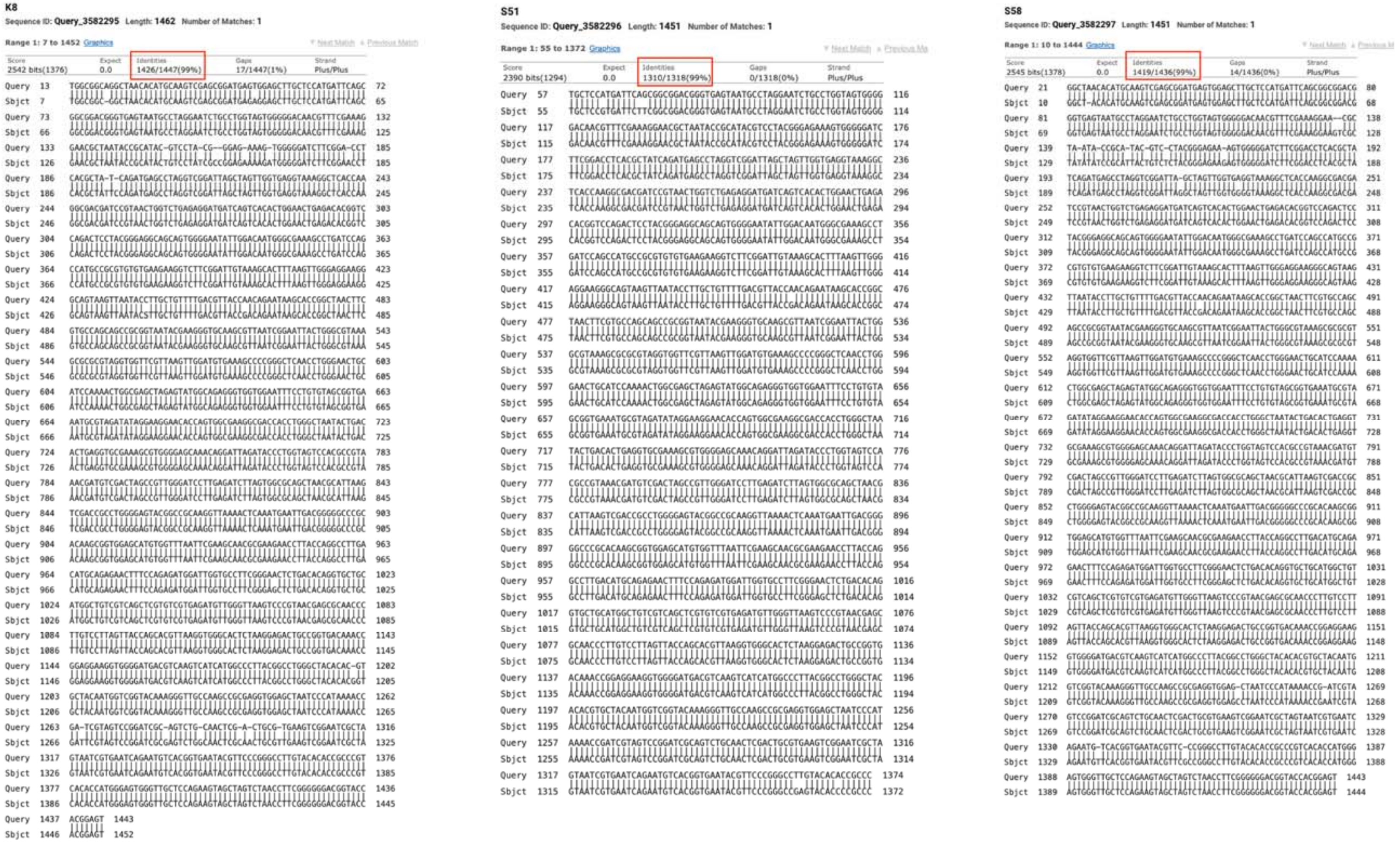

Supplementary Fig. 13 Alignment of S51, S58, and K8 with *Pseudomonas stutzeri*. KC660146.1, 16S rRNA gene sequence of *P. stutzeri* from NCBI.

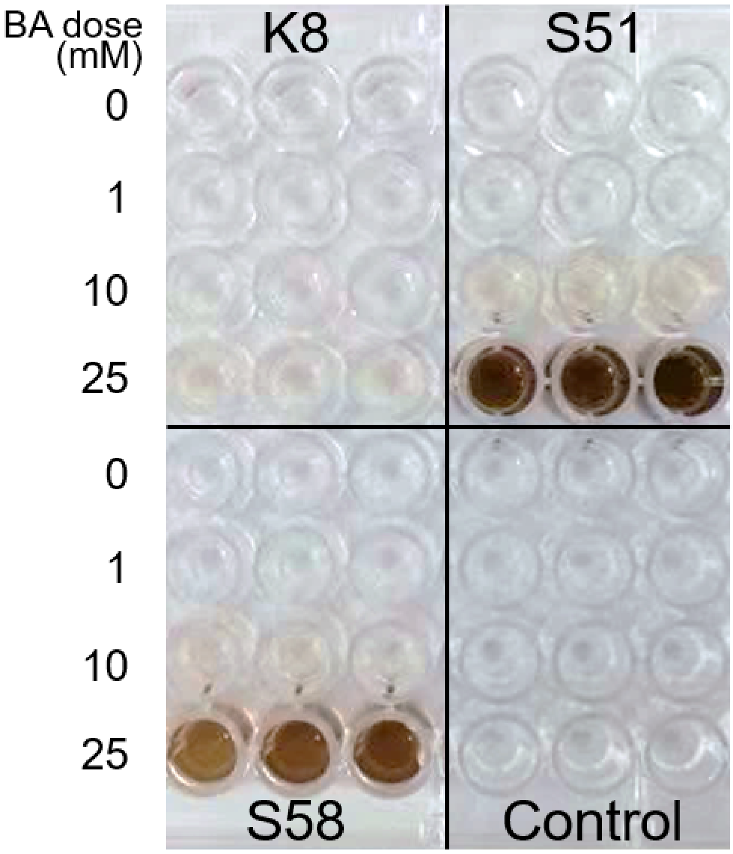

Supplementary Fig. 14 Selected *Pseudomonas* strains use BA as resource. Photograph was taken at the 18th day of culture.

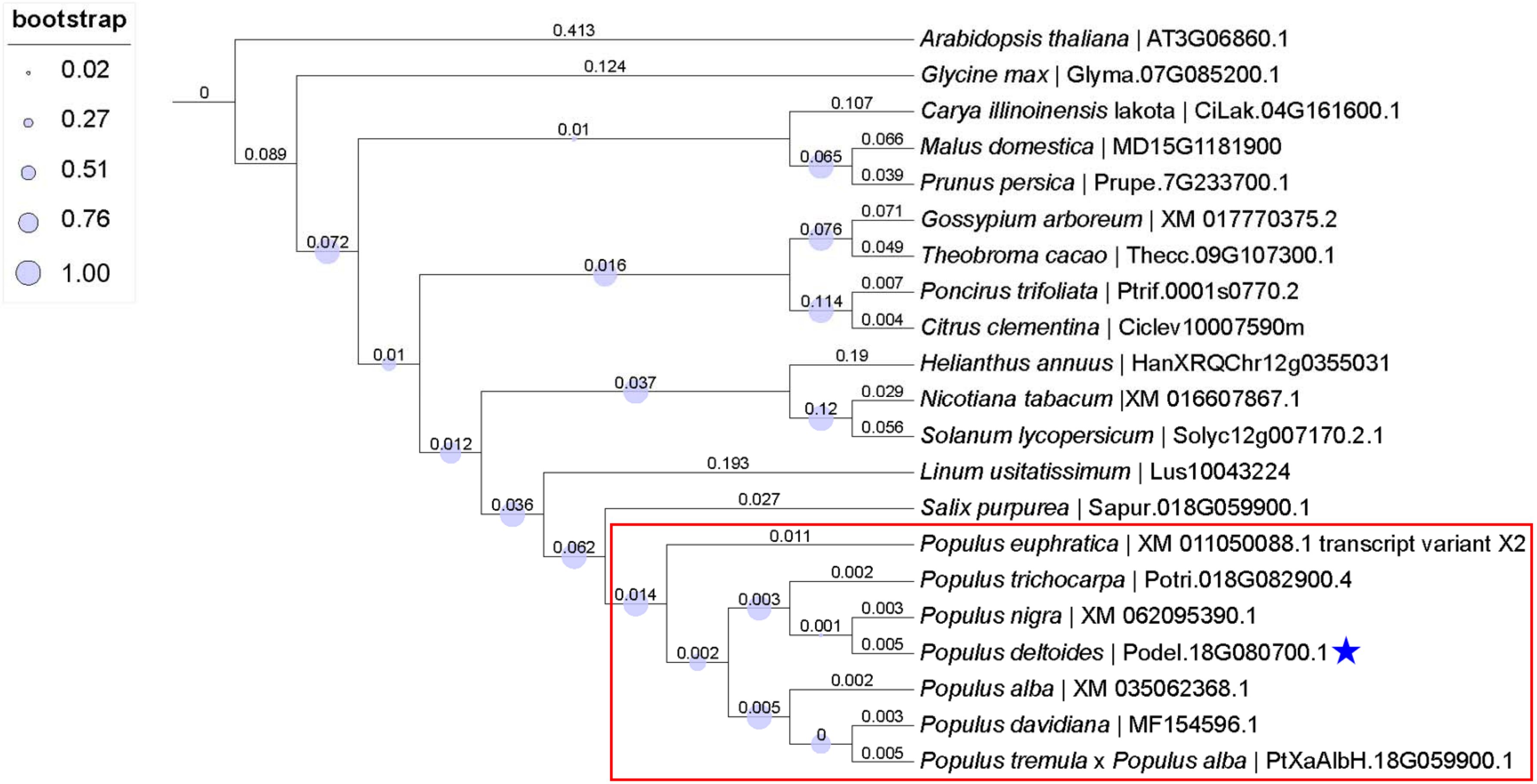

Supplementary Fig. 15 Phylogeny for CDS of *PdaCHD-18g* orthologs from different species. The codes behind "|" indicate the gene number in corresponding databases (Phytozome or NCBI). The numbers stand for genetic distance, species inside the red box come from *Populus* genus, and asterisk indicates the reference of *PdaCHD-18g*.

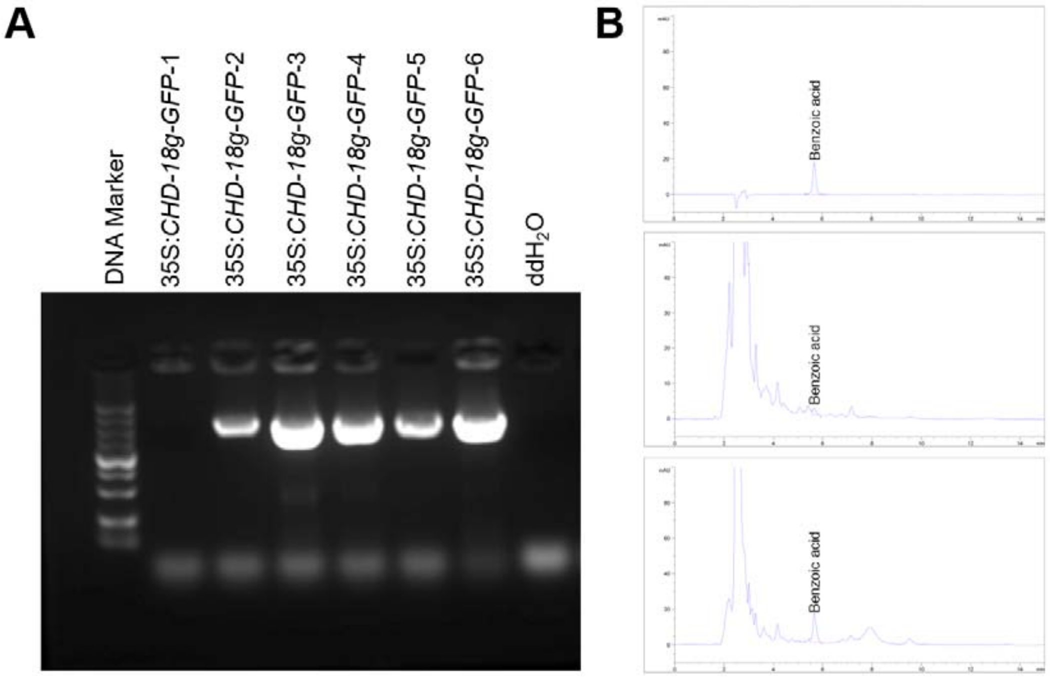

Supplementary Fig. 16 Effect of *PdaCHD-18g* transient over-expression on BA level in tobacco leaves. **A**, DNA identification of pCambia-1300 plasmid recombing *CHD-18g* and *GFP*; and 35S:*CHD-18g-GFP*-2 to 6 were positive clones. **B**, Ion flew of the standard (upper), and samples carrying empty vector (middle) and 35S:*PdaCHD-18g-GFP*-6 (below).

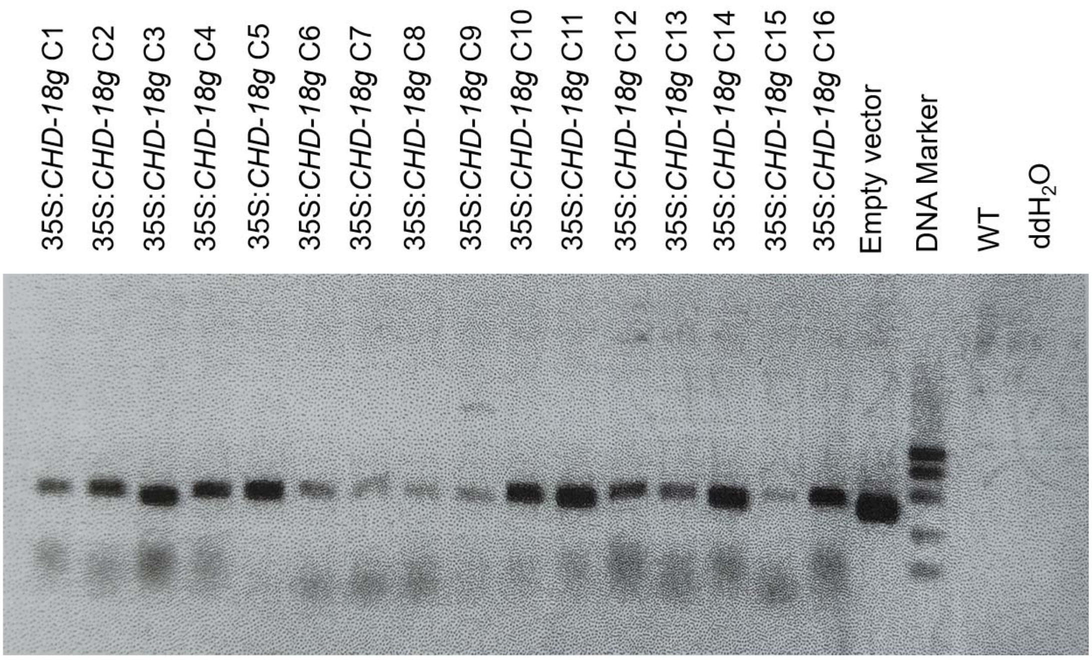

Supplementary Fig. 17 PCR validation of SXY plantlets over-expressing *PdaCHD-18g*. 35S: *PdaCHD-18g* C1 to C16 were positive clones.

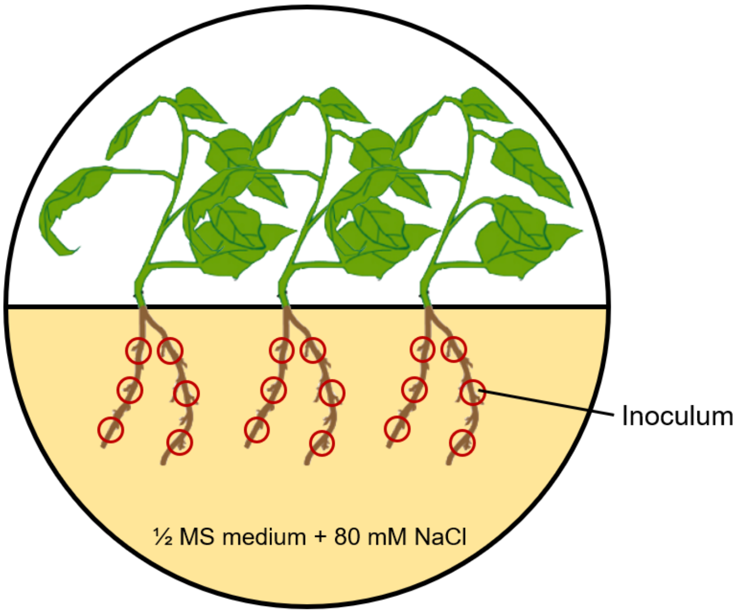

Supplementary Fig. 18 Inoculation of single *Pseudomonas* strain on roots of poplar cultured in MS medium containing NaCl. Sterile ddH_2O is inoculated as control (Mock).

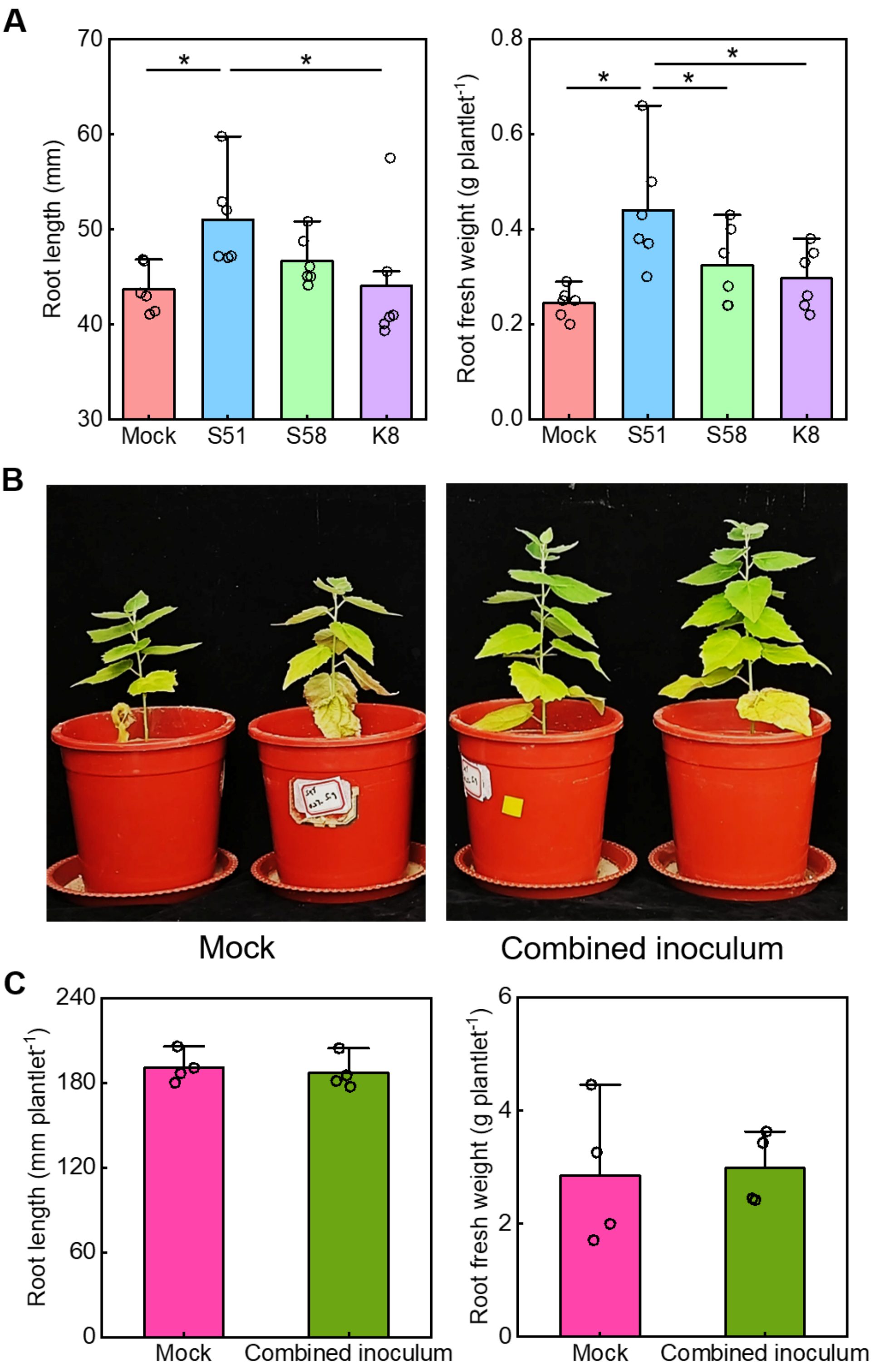

Supplementary Fig. 19 Effect of pseudomonads on SXY under salt stress. **A,** Effect of the selected *Pseudomonas* strains on root biomasses of SXY in NaCl-containing MS medium. Mean ± SD; n = 6 plantlets; and different significance levels between treatments are marked with asterisks (**P* < 0.05, and ***P* < 0.01; ANOVA, LSD). Effect of the combined inoculum on **B**, phenotypes and **C**, root biomass of SXY growing in NaCl-treated sterile natural soil. Mean ± SD; n = 4 plantlets.
